# Uncoupling of nucleo-cytoplasmic RNA export and localization during stress

**DOI:** 10.1093/nar/gkz168

**Published:** 2019-03-13

**Authors:** Hodaya Hochberg-Laufer, Avital Schwed-Gross, Karla M Neugebauer, Yaron Shav-Tal

**Affiliations:** 1 The Mina & Everard Goodman Faculty of Life Sciences & Institute of Nanotechnology, Bar-Ilan University, Ramat Gan 5290002, Israel; 2 Department of Molecular Biophysics and Biochemistry, Yale University, New Haven, CT 06520, USA

## Abstract

Eukaryotic cells contain sub-cellular compartments that are not membrane bound. Some structures are always present, such as nuclear speckles that contain RNA-binding proteins (RBPs) and poly(A)+ RNAs. Others, like cytoplasmic stress granules (SGs) that harbor mRNAs and RBPs, are induced upon stress. When we examined the formation and composition of nuclear speckles during stress induction with tubercidin, an adenosine analogue previously shown to affect nuclear speckle composition, we unexpectedly found that it also led to the formation of SGs and to the inhibition of several crucial steps of RNA metabolism in cells, thereby serving as a potent inhibitor of the gene expression pathway. Although transcription and splicing persisted under this stress, RBPs and mRNAs were mislocalized in the nucleus and cytoplasm. Specifically, lncRNA and RBP localization to nuclear speckles was disrupted, exon junction complex (EJC) recruitment to mRNA was reduced, mRNA export was obstructed, and cytoplasmic poly(A)+ RNAs localized in SGs. Furthermore, nuclear proteins that participate in mRNA export, such as nucleoporins and mRNA export adaptors, were mislocalized to SGs. This study reveals structural aspects of granule assembly in cells, and describes how the flow of RNA from the nucleus to the cytoplasm is severed under stress.

## INTRODUCTION

Originally termed interchromatin granule clusters (IGCs) due to the observation of 20–25 nm nuclear granules in electron microscopy micrographs (1), nuclear speckles are dynamic nuclear bodies (∼20–30 per nucleus) that are enriched in RNA binding proteins (RBPs) (2,3). Prominent examples include the SR proteins with roles in pre-mRNA splicing, mRNA export, RNA stability and translation (4–7). The reasons for RBP accumulation in nuclear speckles and the role these structures play in cells are not completely understood. Most pre-mRNA splicing takes place co-transcriptionally, leading to the proposal that nuclear speckles are sites of assembly and/or storage of the pre-mRNA splicing machinery. Because RBPs are not retained in speckles, they are able to diffuse to active sites of transcription, where they participate in splicing. Alternatively, they may be recruited from nuclear speckles to pre-mRNA in the nucleoplasm during post-transcriptional splicing, mRNP formation and/or mRNA export (2,8,9).

While nuclear speckles contain a large variety of splicing factors, they are also enriched in poly(A)+ RNAs comprised of both and long non-coding RNAs (lncRNAs) and mRNAs (10,11). Whether these RNA components participate in the formation of nuclear speckles and their maintenance remains unclear. For instance, the localization of several SR splicing factors to nuclear speckles in cancer cells required interactions with MALAT1 (metastasis-associated lung adenocarcinoma transcript 1), a lncRNA that specifically localizes to nuclear speckles (12); however, MALAT1 did not seem to influence the localization of nuclear speckle components in mouse tissues (13). Thus, MALAT1 is not thought to be necessary for nuclear speckle formation or maintenance (10).

Recently, a library of natural compounds was screened in search of molecules that affect nuclear speckle structure and gene expression (14). Nuclear speckles were identified in the screen using poly(A)+ RNA fluorescence *in situ* hybridization (FISH) with a fluorescent oligo-dT probe that hybridized with the poly(A) tails. Tubercidin, an adenosine analogue in which the N-7 position of adenosine is replaced with C–H (7-deaza-adenosine), caused the complete delocalization of poly(A)+ RNA from nuclear speckles after 1 hr of treatment in HeLa cells. This was accompanied by the dispersal of the splicing factor SRSF1 from nuclear speckles. However, nuclear speckles were not disassembled, and the splicing factor SRSF2 remained within them, accompanied by changes in their overall structure.

Tubercidin was reported initially as having antibiotic and anti-cancer effects (15). This analogue of adenosine was identified from culture filtrates of *Streptomyces tubercidicus*, and held promise for treatment in the clinic. Tubercidin is incorporated into DNA and RNA and was shown to negatively affect protein synthesis (16). The cytotoxic effects of tubercidin on cancer cells drove experiments that tested its safety in animals (mice, rats, dogs and monkeys) (17) and later in humans. Altogether, phase I trials concluded that treating humans with tubercidin is inadequate due to severe toxicity (18–20).

The mechanistic explanation(s) for the high levels of tubercidin toxicity on normal cells was not explored. Its ability to incorporate into DNA, RNA and affect protein synthesis have been briefly documented and does not explain why this compound had such extreme effects in comparison to other adenosine analogues. With respect to nuclear speckles, it was suggested that tubercidin's incorporation into RNA may lead to the dispersal of RNA from nuclear speckles and perhaps to reduced mRNA stability (14). In our study, we set out to investigate the formation and composition of nuclear speckles and the fate of the RNAs and proteins within them, using tubercidin as a tool that compromises nuclear speckle structure. However, we discovered that tubercidin effects were much broader than anticipated, generating a cellular stress response. Although transcription and splicing continued to transpire, the stress conditions inhibited mRNP formation and mRNA export in multiple cell types. We found that many nuclear proteins and RNAs were mislocalized under these stress conditions, particularly components of the nuclear pore complex (NPC) and RNA processing factors that are necessary for mRNA export. All these defects hindered the nucleo-cytoplasmic flow required for the gene expression pathway. Moreover, this analysis has enabled us to examine the structural aspects of RNA-protein granules in cells during their assembly and disassembly. Finally, the induction of this stress response by tubercidin in normal and cancer cells can explain why this compound failed as a chemotherapeutic agent.

## MATERIALS AND METHODS

### Plasmids

IGF2BP3-GFP was received from S. Hüttelmaier (21) and GFP-CF I_m_68 and GFP-CF I_m_25 were received from S. Barabino (22). GFP-Dbp5 was received from S. Wente (23). For the plasmid encoding HIST1H2BA, the gene was amplified by RT-PCR (GoTaq Green Mix, Biological Industries) from genomic DNA (TIANamp Genomic DNA kit, TIANGEN) of U2OS cells, and subcloned into peGFP-C1 using primers containing the restriction enzyme sites of BsrGI and HindIII:

Forward- ATATGTACATTCCCTCGTCCAGACATCTCCT.
Reverse- TATAAGCTTTGGGATTACAGGCGAGTCACGG.

### Cells and transfections

Human U2OS cells were maintained in low glucose Dulbecco's modified Eagle's medium (DMEM, Biological Industries, Israel) containing 10% fetal bovine serum (FBS, HyClone). The cell lines HeLa, mouse embryonic fibroblasts (MEF) (24), human foreskin fibroblasts (hFF), HCT116, PANC1, SW620 and MCF7 were maintained in high glucose DMEM containing 10% FBS. Transgenic U2OS E3 and E6 cell lines (25) with stable integration of BACs carrying C-terminally tagged SRSF2, SRSF4, SRSF5, SRSF6, SRSF7, U1-70K, U2AF65 and Prp8, were generated as described (26,27). The U2OS cell line expressing CFP-actin and the MEF β-actin-MS2 cells were previously described (24,28).

Stress granules were formed by adding arsenite (1 Mm, Sigma) to the medium for up to 45 min or sorbitol (600 mM, Sigma) for 1.5 hr. Tubercidin (10 μM, Sigma), toyocamycin (10 μM, Sigma) and adenosine (10 μM, Sigma) were added to the medium for different times. For inhibition of SG assembly, cells were treated for 3 hrs with GSK2606414 (10 μM, Cayman) before tubercidin addition. Cells were induced to express the β-globin-MS2 by the addition of 1 μg/ml doxycycline (Sigma). For splicing inhibition, cells were treated for 6 hrs with Pladienolide B (10 μM, Santa Cruz). For transcription inhibition, cells were treated for 2 h with actinomycin D (ActD) (5 μg/ml, Sigma).

The Click-iT^®^ RNA Alexa Fluor^®^ 488 Imaging Kit (Invitrogen) was used for global RNA visualization. Cells were treated with the uridine analog 5-ethynyluridine (1 mM EU) only or together with tubercidin for 3 h. Then the EU was washed and fresh medium with or without tubercidin was added to the cells for another 3 h. After fixation, the Click-iT reaction cocktail was added to the cells for 30 min and washed with the Click-iT reaction rinse buffer.

For transient transfections, cells were transfected with 1–5 μg of plasmid DNA and 40 μg of sheared salmon sperm DNA (Sigma) when using electroporation (Gene Pulser Xcell, Bio-Rad). Stable expression of IGF2BP3-GFP was obtained by co-transfection of the cells with IGF2BP3-GFP (10 μg) and puromycin resistance (300 ng) plasmids using electroporation (Gene Pulser Xcell, Bio-Rad) and selection with puromycin (1 μg/ml; Invivogen, San Diego, CA). An E6 stable cell line expressing YFP-MS2 was created as described in (29).

### Western blotting

Cells were washed with cold PBS and placed on ice for 15 min after resuspending in immunoprecipitation (IP) lysis buffer (Pierce) containing 10 mM Na-fluoride, 1 mM Na-orthovanadate, protease inhibitor cocktail (Sigma) and 1 mM phenylmethylsulfonyl fluoride (PMSF). The resulting lysate was centrifuged at 10,000 rpm for 10 min at 4°C. 30  μg of protein was run on SDS-polyacrylamide gels and transferred to a nitrocellulose membrane (0.45 μm). The membrane was blocked with 5% BSA and then probed with a primary antibody for 2 h at room temperature (RT), followed by incubation with HRP-conjugated goat anti-rabbit/mouse IgG (Sigma) for 1 h at RT. Immunoreactive bands were detected by the Enhanced Chemiluminescence kit (ECL, Pierce). Primary antibodies used were rabbit anti-eIF2α (Abcam), rabbit anti-P-eIF2α, mouse anti-GFP and rabbit anti-tubulin. Experiments were performed three times and quantified. The quantification of the blots was performed in ImageJ. After the selection of the bands, the values of the intensities were obtained. The intensities of the eIF2α and P-eIF2α bands were divided by the corresponding tubulin band intensities.

### Immunofluorescence

Cells were grown on coverslips, washed with PBS and fixed for 20 min in 4% PFA. Cells were then permeabilized in 0.5% Triton X-100 for 2.5 min. Cells were washed twice with PBS and blocked with 5% BSA for 20 min cells and immunostained for 1 h with a primary antibody. After three washes with PBS, the cells were incubated for 1 h with secondary fluorescent antibodies. Primary antibodies: mouse anti-G3BP1 (Abcam), rabbit anti-hnRNP A1, rabbit anti-eIF2α, rabbit anti-eIF4B, rabbit anti-CBP80, rabbit anti-U2AF65, rabbit anti-eIF4A3, mouse anti-Y14, mouse anti-Aly, mouse anti-UAP56, rabbit anti-Dbp5, rabbit anti-Nup153, mouse anti-NXF1, rabbit anti-Nup214, rabbit anti-Nup358, mouse anti-Nup62, rat anti-Nup98, rabbit anti-TPR, rabbit anti-THOC5, goat anti-TIA1 (Santa Cruz), mouse anti-CBP20, mouse anti-THOC6, mouse anti-SC35 (Sigma), mouse anti-RACK1 (BD Biosciences). Secondary antibodies: Alexa488-labeled goat anti-mouse/rabbit/rat IgG (Abcam), Alexa488-labeled donkey anti-goat IgG, Alexa594-labeled goat anti-mouse/rabbit. Nuclei were counterstained with Hoechst 33342 (Sigma) and coverslips were mounted in mounting medium. For colocalization analysis, the coloc2 ImageJ plugin was used to calculate Pearson's correlation coefficient (*R*_r_) to measure the colocalization degree. One sample *t*-test was performed.

### Fluorescence *in situ* hybridization

Cells were grown on coverslips and fixed for 20 min in 4% paraformaldehyde, and overnight with 70% ethanol at 4°C. The next day cells were washed with 1× PBS and treated for 2.5 min with 0.5% Triton X-100. Cells were washed with 1× PBS and incubated for 10 min in 40% formamide (4% SSC; Sigma). Cells were hybridized overnight at 37°C in 40% formamide with a specific fluorescently-labeled Cy3 or Cy5 DNA probe (∼10 ng probe, 50 mer). The next day, cells were washed twice with 40% formamide for 15 min and then washed for two hours with 1× PBS. For oligo-dT labeling, 15% formamide was used. Nuclei were counterstained with Hoechst 33342 and coverslips were mounted in mounting medium. The probe for the MS2 binding site was: CTAGGCAATTAGGTACCTTAGGATCTAATGAACCCGGGAATACTGCAGAC. PolyA: an oligo(dT) probe. The intron probe was from (25). In some cases, immunofluorescence was performed after the RNA FISH using the standard protocol.

FISH experiments with Stellaris (Biosearch Technologies) probes were performed according to the manufacturer's adherent cell protocol. Probes used were: CCND1, ANLN, HIST1H2BA, MALAT1 and NEAT1. To reduce photobleaching, the slides were mounted in GLOX buffer (pH 8,10 mM, 2× SSC, 0.4% glucose), supplemented with 3.7 ng of glucose oxidase (Sigma G2133-10KU) and 1 μl Catalase (Sigma 3515) prior to imaging.

### Fluorescence microscopy and live-cell imaging

Wide-field fluorescence images were obtained using the Cell^∧^R system based on an Olympus IX81 fully motorized inverted microscope (60× PlanApo objective, 1.42 NA) fitted with an Orca-AG CCD camera (Hamamatsu) driven by the Cell^®^ software. Live-cell imaging was carried out using the Cell^∧^R system. For time-lapse imaging, cells were plated on glass-bottomed tissue culture plates (Greiner Bio-One GmbH, Germany) in medium containing 10% FBS at 37°C. The microscope is equipped with an incubator that includes temperature and CO_2_ control (Life Imaging Services, Reinach, Switzerland). For long-term imaging, several cell positions were chosen and recorded by a motorized stage (Scan IM, Märzhäuser, Wetzlar-Steindorf, Germany). Cells were imaged immediately after the treatment with tubercidin or arsenite in 3D every 15 min, at 1 μm steps for 6 h. For the presentation of the movies, the 4D image sequences were transformed into a time sequence using the maximum or sum projection options or manually selecting the in-focus plane using the ImageJ software.

### RT-PCR

Total RNA was extracted using Tri-Reagent (Sigma) and DNA removed using a Turbo-DNase free kit (Ambion). cDNA (1 μg RNA) was synthesized using the ReverseAid First Strand cDNA Synthesis Kit (Fermentase) and PCR was performed using GoTaq green mix (Promega) with the following primers:

E6 forward: TCTGACACAACTGTGTTCAC
E6 reverse: TCCACGTGCAGCTTGTCACA
p27 forward: CAGCTTGCCCGAGTTCTACT
p27 reverse: GTCCATTCCATGAAGTCAGCG
DXO forward: TGGGGAGGTTAACACCAACG
DXO reverse: GCTCTGGGAAAGCTA AGGA
MCL1 forward: GAGGAGGAGGAGGACGAGTT
MCL1 reverse: ACCAGCTCCTACTCCAGCAA
NOP56 forward: GCATCCACAGTGCAGATCCT
NOP56 reverse: GCAATCGATTCGTGAGGCAA
HSP40 forward: GAACCAAAATCACTTTCCCCAAGGAAGG
HSP40 reverse: AATGAGGTCCCCACGTTTCTCGGGTGT

### Statistical analysis

The intensities of P-eIF2α and eIF2α in the western blots were log_2_-transformed and the effect of tubercidin, arsenite, toyocamycin and adenosine treatments for different time points was compared to the control with a one-sample *t*-test against a mean of 0. All the experiments were repeated at least three times. The results are presented as mean values ± SEM of three independent experiments.

## RESULTS

### Differential effect of tubercidin on RNAs and nuclear speckle proteins

**Figure 1.**
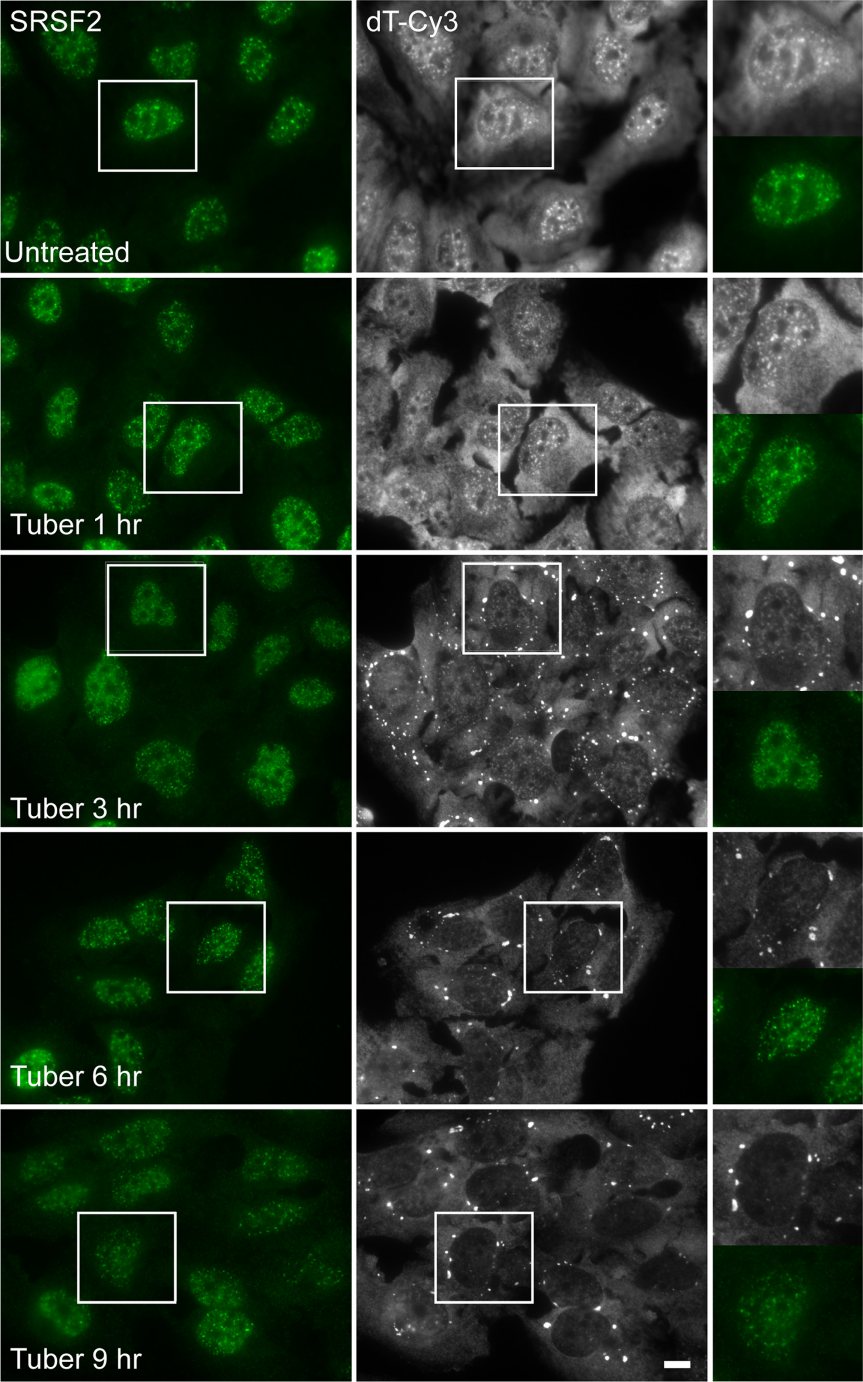
Tubercidin affects the sub-cellular distribution of poly(A)+ RNA. The distribution of poly(A)+ RNA was examined in U2OS cells treated with tubercidin for several hours. Poly(A)+ RNA was detected using RNA FISH with an oligo-dT probe (gray), while nuclear speckles were detected by immunofluorescence using an antibody to SRSF2 (green). Enlarged areas are shown on the right. Bar = 5 μm.

To examine the effect of tubercidin treatment over time on poly(A)+ RNAs as well as on nuclear speckle proteins, we treated U2OS cells with tubercidin for several hours. The distribution of the splicing factor SRSF2 that localizes to nuclear speckles was examined using immunofluorescence whereas poly(A)+ RNA, also normally accumulating in nuclear speckles, was detected by RNA fluorescence *in situ* hybridization (FISH) with a fluorescent oligo-dT probe. Although the continued presence of nuclear speckles was observed, as seen by SRSF2 staining, the poly(A)+ RNA population redistributed from the nucleus and accumulated in cytoplasmic structures (Figure 1). Since tubercidin is a toxic adenosine analogue that interferes with translation, we considered the possibility that it may be lead to stress granule (SG) formation, cytoplasmic structures that are induced upon several cellular stresses and contain untranslated mRNAs and various RBPs (30,31).

**Figure 2.**
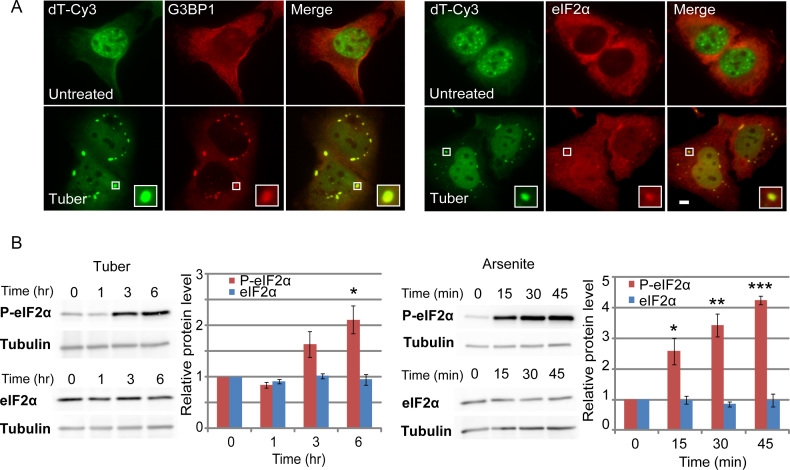
mRNAs accumulate in SGs during tubercidin treatment. (**A**) U2OS cells treated with tubercidin (6 hrs) show the translocation of poly(A)+ RNAs detected by RNA FISH (oligo-dT probe, green) into SGs labeled with anti-G3BP1 and eIF2α antibodies (red). Boxed regions in the images are shown in enlarged boxes. Bar = 5 μm. (**B**) Tubercidin increases phosphorylated eIF2α levels. Western blot analysis of eIF2α and phosphorylated eIF2α protein levels in U2OS cells after tubercidin or arsenite treatments for different times. Tubulin was used as a loading control. Blots are representative of three independent experiments. The average quantification of three repeated experiments is presented in the plots (mean ± SEM). There were significant differences in the relative levels of phosphorylated eIF2α between the times point 0 h and 6 h in cells treated with tubercidin (one sample *t*-test, *P* = 0.03141) and between 0 h and 15, 30 and 45 min in cells treated with arsenite (one sample *t*-test, *P* = 0.033, *P* = 0.007246, *P* = 0.0004958). ***P < 0.001, **P < 0.01, *P < 0.05.

To determine whether mRNAs accumulated in SGs under tubercidin treatment, we examined whether the poly(A)+ RNA structures colocalized with SGs markers. Indeed, the strong RNA signal was found to accumulate in SGs after tubercidin treatment (6 h; Figure 2A). Tubercidin caused SG formation in different cancer cell lines (HeLa, MCF7), in normal human foreskin fibroblast cells (hFFs) and in mouse embryonic fibroblasts (MEFs) (data not shown). Since the composition of SGs can vary, we examined the presence of several SG protein markers – G3BP1 (GAP SH3 Domain-Binding Protein 1), TIA1 (T-Cell-Restricted Intracellular Antigen-1), hnRNP A1 (Heterogeneous Nuclear Ribonucleoprotein A1), eIF2α (Eukaryotic Translation Initiation Factor 2A), eIF4B (Eukaryotic Translation Initiation Factor 4B). All these proteins accumulated in SGs (Figure 2A and data not shown). Since the classic stress pathway usually includes the phosphorylation of eIF2α as part of the translation inhibition taking place during stress, we examined whether tubercidin also activated this pathway. Indeed, increased detection of phosphorylated eIF2α was observed upon either arsenite, the classical inducer of SGs, or tubercidin treatment (Figure 2B). However, the effect occurred within minutes of arsenite treatment, whereas eIF2α phosphorylation was seen only after several hours of tubercidin treatment. To independently address differences in the time course of the tubercidin stress response, time-lapse imaging of U2OS cells stably expressing GFP-IGF2BP3 was performed. Visible SGs were detected after 15 min in arsenite, while SG formation took ∼90 min in tubercidin treated cells (Supplementary Movies S1 and S2). Interestingly, the adenosine analogue toyocamycin, a close relative of tubercidin, did not have any of the above effects, nor did adenosine itself (Supplementary Figure S1).

### Tubercidin induces RNA accumulation in stress granules

**Figure 3.**
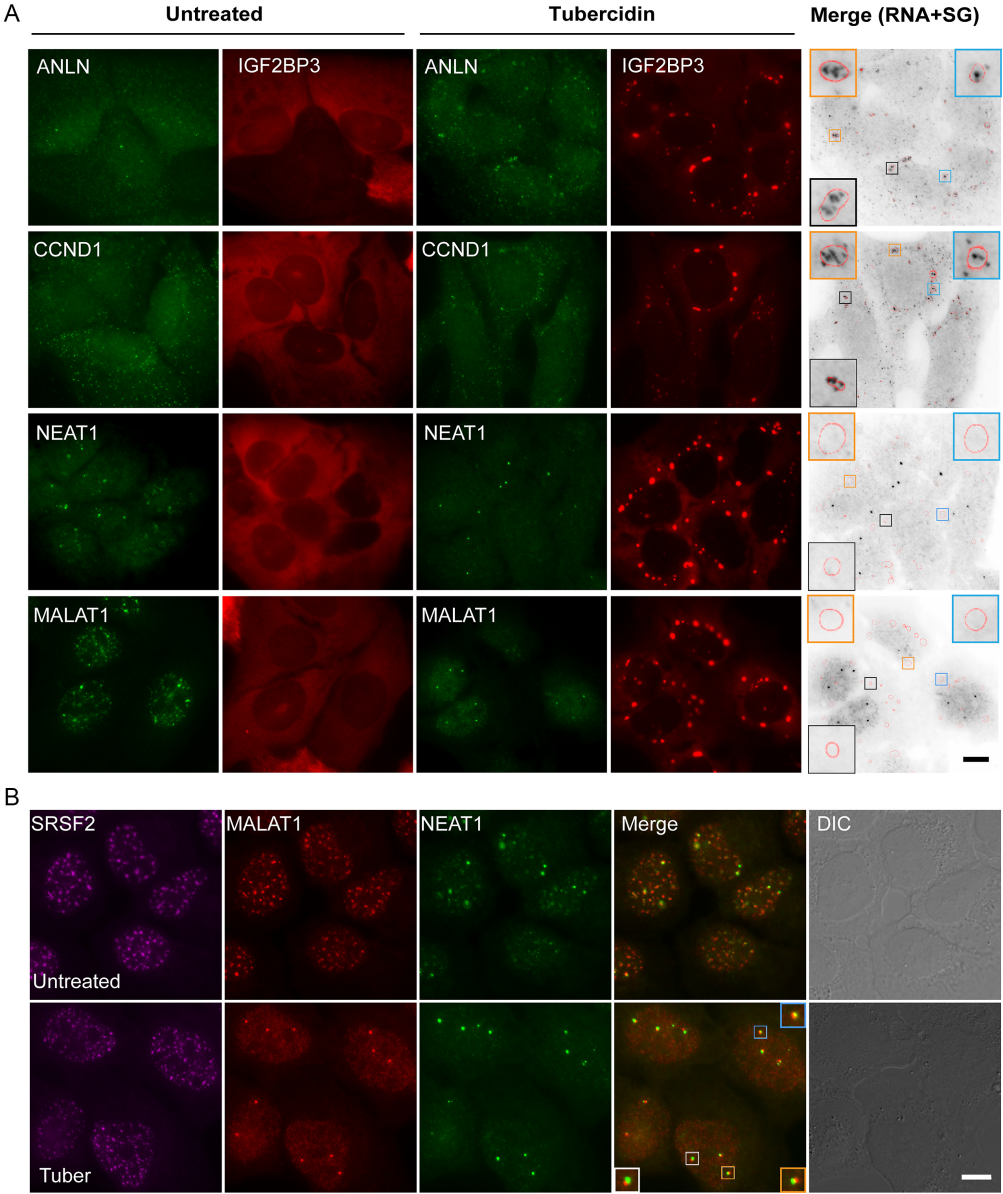
Accumulation of different RNAs in SGs following tubercidin treatment. (**A**) The localization of ANLN and CCND1 mRNAs, and NEAT1 and MALAT1 lncRNAs under tubercidin treatment (6 h) detected using RNA FISH with specific probes to these endogenous RNAs (green). SGs were detected by expression of GFP-IGF2BP3 (pseudo-colored red). The grey image is an inverted version of the RNA FISH images (green), emphasizing the localization of mRNAs (black) in SGs (SG periphery is marked in red in the boxed SGs). Bar = 10 μm. (**B**) Tubericidin does not interfere with MALAT1 and NEAT1 transcription. RNA FISH with probes to MALAT1 (red) and NEAT1 (green) show that these lncRNAs disperse in the nucleoplasm during tubercidin treatment, and do not localize to nuclear speckles (anti-SRSF2, magenta). Three MALAT1 and NEAT1 active transcription sites overlap (merge). Bar = 10 μm.

RNA FISH experiments with a fluorescent oligo-dT probe can be used to detect both coding and non-coding RNA molecules. In order to examine the localization of specific RNAs during tubercidin treatment, we used specific probes to several endogenous RNAs. The endogenous anillin (*ANLN*) and cyclin D1 (*CCND1*) mRNAs were partially localized to SGs (Figure 3A), as previously shown (32). Two nuclear-retained lncRNAs—MALAT1 normally located in nuclear speckles and NEAT1 located in the paraspeckles—were not detected in cytoplasmic SGs and were dispersed in the nucleoplasm upon tubercidin treatment (Figure 3A, B). Nevertheless, three fluorescent foci, which we postulated were the transcribing alleles of *MALAT1* and *NEAT1*, remained detectable in the nucleus.

U2OS cells are expected to have three copies of chromosome 11 (33) and therefore three *MALAT1* alleles. Since the *MALAT1* gene is situated 50 kb from the gene to *NEAT1* (34), we assumed that a probe to the NEAT1 lncRNA should colocalize with the transcribing loci of *MALAT1*. Indeed, three NEAT1 foci were found in partial colocalization with the three MALAT1 foci (Figure 3B), suggesting that both genes continued to transcribe under tubercidin treatment. To further validate that the foci represented the active genes transcribing MALAT1 and NEAT1, we used additional cell types harboring different copy numbers of chromosome 11. Two foci were detected in normal human foreskin fibroblast cells (hFFs) and in human colon cancer cells HCT116, three foci in human breast cancer MCF7 and human colon cancer SW620 cells, and four in human pancreatic cancer PANC1 cells (Supplementary Figure S2). Finally, transcription inhibition by actinomycin D eliminated the three foci, and MALAT1 distributed in the nucleoplasm while nuclear speckles remained (Supplementary Figure S3A). This suggests that MALAT1 and NEAT1 lncRNAs are not essential for nuclear speckle formation and maintenance, and that tubercidin does not inhibit transcription.

### Tubercidin does not inhibit transcription but impairs nuclear mRNA export

To examine which points in the gene expression pathway are targeted during stress, we monitored the entire gene expression pathway for a single expressed gene, employing an inducible reporter gene termed E6 (25). The E6 gene is based on a Tet-inducible β-globin mini-gene and contains six exons and five introns. The coding region is N-terminally fused to a cyan fluorescent protein (CFP) containing a SKL tri-peptide that targets the protein to cytoplasmic peroxisomes upon doxycycline (dox) induction. The E6 mRNA also contains a series of MS2 sequence repeats in its 3′UTR (35). A probe complementary to the MS2 sequence repeats allows robust detection of the E6 mRNA by RNA FISH (Supplementary Figures S3 and S4A). Upon tubercidin treatment, the E6 mRNA was detected in SGs as well as in the nucleus (Supplementary Figure S4A). The CFP protein product was not detected (Supplementary Figure S4B, C), confirming stress-induced translation inhibition (36).

**Figure 4.**
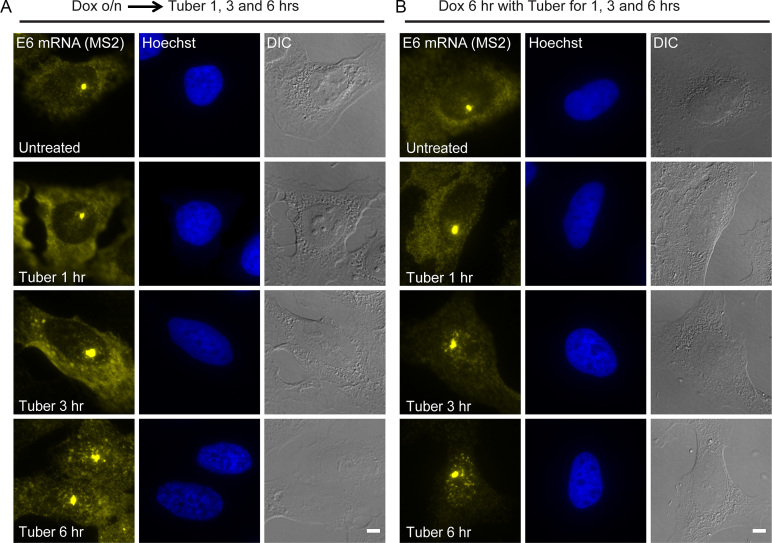
Tubercidin does not inhibit transcription but inhibits nuclear mRNA export. E6 mRNA was detected after tubercidin treatment for different times, using RNA FISH with a probe to the MS2 region in the E6 mRNA (yellow). Tubercidin was added either (**A**) after overnight (o/n) induction of the E6 gene with doxycycline, or (**B**) together with the dox induction for 6 h. Hoechst DNA stain is in blue. Bar = 5 μm.

The E6 mRNA is usually predominantly cytoplasmic. Interestingly, E6 mRNA accumulated in the nucleus upon tubercidin treatment (Supplementary Figure S3). Since the E6 gene is transcriptionally inducible, we could determine the effect of tubercidin on E6 mRNA fate by varying the timing of dox and tubercidin treatments, and then examine whether tubercidin has an effect on nucleo-cytoplasmic mRNA transport. After overnight dox induction during which the E6 transcripts are transcribed, tubercidin was added for 6 h. The E6 mRNA was abundantly detected at the sites of transcription, which also persisted after subsequent tubercidin treatment, and the mRNAs were observed in the cytoplasm but also accumulated in the nucleus (Figure 4A). These results suggested that tubercidin did not inhibit transcription, in comparison to actinomycin D transcriptional inhibition that eliminated the site of the active gene (Supplementary Figure S3).

The nuclear accumulation of the E6 mRNA suggested a possible obstruction of mRNA export. To test this, tubercidin was administered to the cells together with dox induction (Figure 4B). This form of the experiment allowed us to discriminate between the transcripts that were transcribed before or during tubercidin treatment. At the early time points, E6 mRNAs were detectable in the cytoplasm; however, at later time points, the cytoplasmic levels of the E6 mRNA were very low and the mRNAs accumulated in nuclear puncta. This implied impaired mRNA export. Namely, the cytoplasmic transcripts in stress granules had probably been transcribed prior to the tubercidin treatment, while the nuclear retained mRNAs apparently represent transcripts transcribed during the tubercidin treatment.

**Figure 5.**
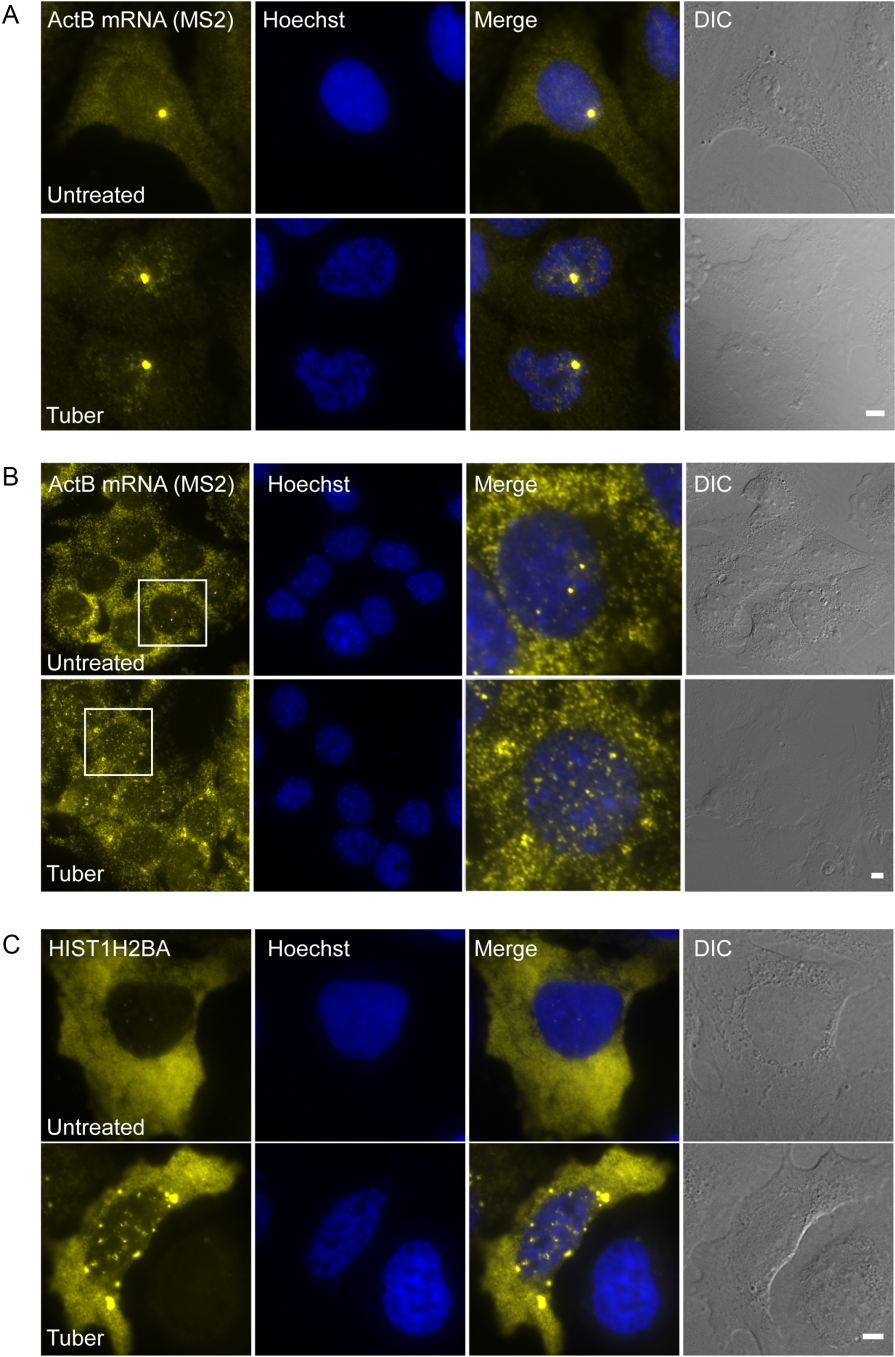
Tubercidin impairs mRNA export of endogenous and exogenous β-actin mRNAs and histone non-polyadenylated mRNAs. (**A**) Dox-induced β-actin mRNAs were detected in U2OS cells after tubercidin treatment for 6 h and (**B**) endogenous β-actin mRNAs were detected in MEFs after tubercidin treatment for 3 h using RNA FISH with a probe to the MS2 region in the 3′UTR of the mRNAs (yellow). Big yellow dot in A and strong dots in B are transcription sites. Hoechst DNA stain is in blue. Boxed regions are shown in the merge. Bar = 5 μm. (**C**) Histone H2B mRNAs were detected in U2OS cells after tubercidin treatment for 6 h using RNA FISH with a probe to the coding region (yellow). Hoechst DNA stain is in blue. Bar = 5 μm.

To examine whether the mRNA export impairment affects other transcripts, we used two additional inducible mRNA systems that contain MS2 repeats and allow control of gene expression timing relative to tubercidin administration. The two mRNAs contain MS2 sequences in their 3′UTR and were detected by RNA FISH with a probe to this region. Both a dox-inducible CFP-β-actin mRNA (28) (Figure 5A) and endogenous β-actin mRNAs in MEFs (24) (Figure 5B), the latter induced to transcribe by serum induction (37), demonstrated that the mRNAs remained stuck in the nucleus after tubercidin treatment. In addition, we demonstrated that histone H2B mRNAs, which have a different 3′-end processing mechanism (38), were also stuck in the nucleus upon tubercidin treatment (Figure 5C).

**Figure 6.**
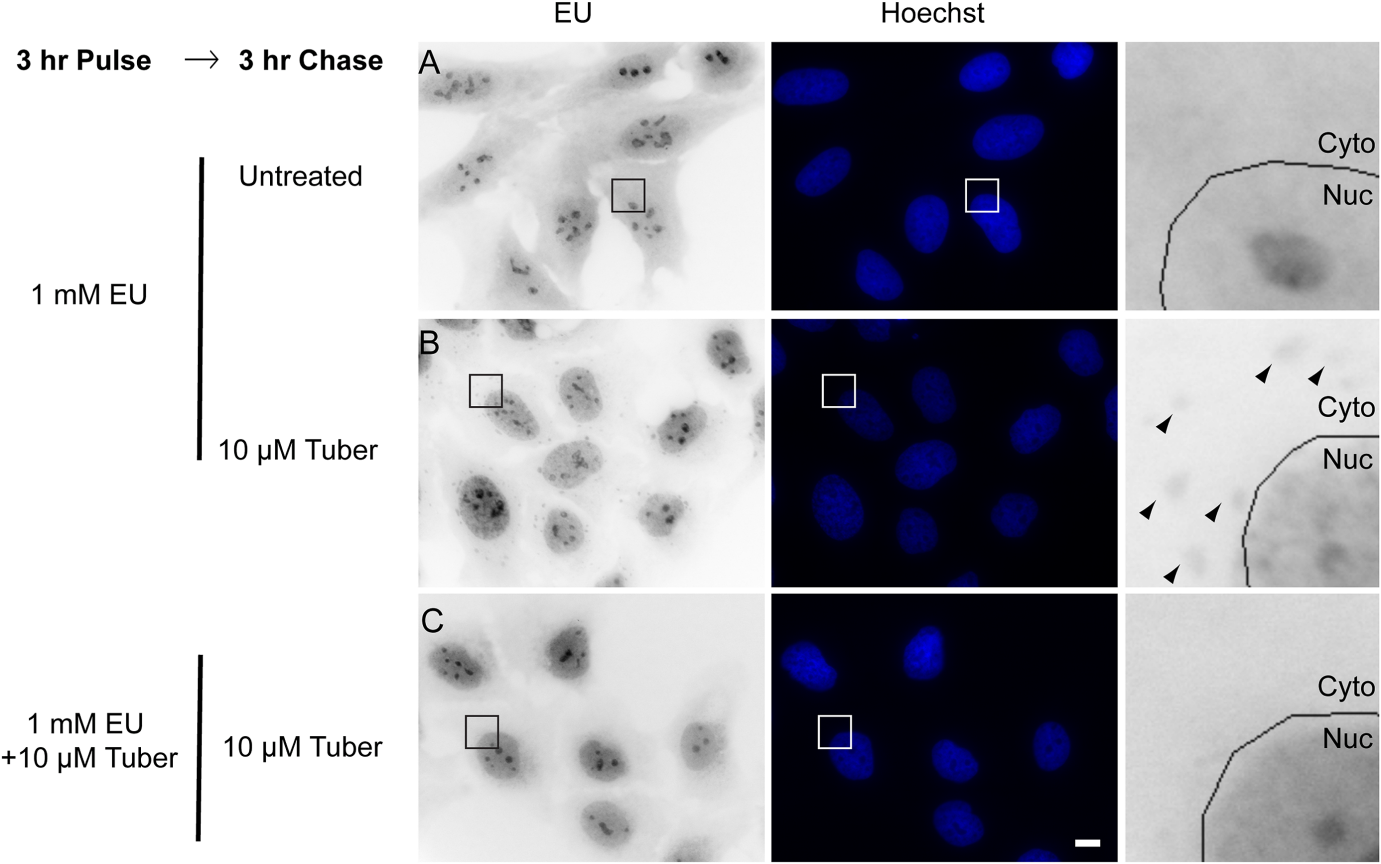
Tubercidin has a negative effect on global mRNA export. Cells were treated with the modified nucleotide ethynyl uridine (EU, 1 mM) for 3 h together with and without tubercidin, in order to label cellular RNAs. Then the EU washed and regular medium (**A**) or tubercidin (**B** and **C**) were added for 3 h. In B tubercidin was added after the EU labeling (stress granules are marked with arrowheads) and in C tubercidin was added together with the EU and an increase in the nuclear stain was observed. Enlarged sections are shown in the right hand column. Hoechst DNA stain is in blue. Bar = 10 μm.

Finally, to examine if tubercidin has a global effect on mRNA export, we used the modified nucleotide 5-ethynyluridine (EU) that stains RNA in cells (39), in order to follow global RNA export (Figure 6). The experiment was conducted in two ways in order to discriminate between new and old transcripts: (i) EU was added to the cells and allowed to incorporate into RNA for 3 h. This allows enough time for mRNAs to be exported into the cytoplasm (Figure 6A). Three hours later tubercidin was added and the accumulation of EU-tagged RNA in stress granules was observed, as was mRNA accumulation in the nucleus (Figure 6B). (ii) EU was added together with tubercidin (Figure 6C) and most of the EU-tagged transcripts were not able to exit the nucleus (increase in the nuclear stain), and were not seen in the cytoplasmic stress granules. The significant nuclear accumulation of mRNA that was observed implies that the export impairment is quite severe causing a global defect in mRNA export.

### Stress redistributes nucleoporins and mRNA export factors into SGs

**Figure 7.**
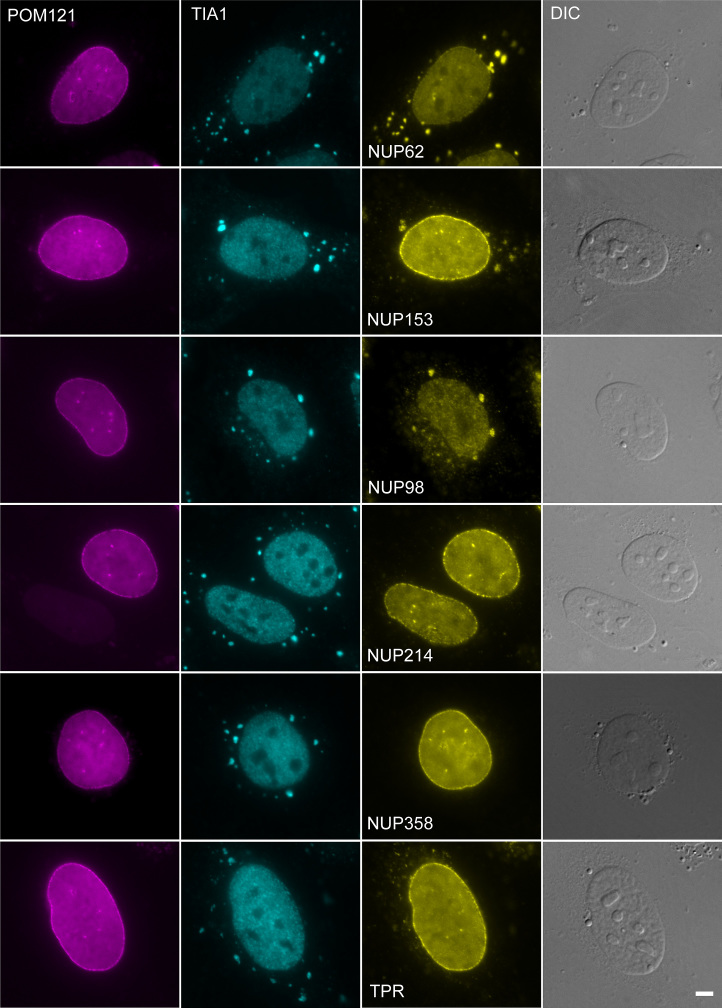
Nucleoporins accumulate in SGs during tubercidin stress. Tubercidin stress (6 h) causes the accumulation of some nucleoporins in SGs. POM121 nucleoporin that does not change during stress was used as a control to demarcate the NPCs. SGs were identified with an antibody to TIA-1. Bar = 5 μm.

We examined whether tubercidin-induced stress might be interfering with components of the mRNA export machinery. First, we examined whether nuclear pore complex (NPC) proteins might be affected. Indeed, the nucleoporins (Nups) Nup62, Nup98 and Nup153 substantially accumulated in the stress granules (Figure 7). Nup153 is located in the nuclear basket and has an important role in the first steps of mRNA export (40–42). Similar redistribution of Nup62 and Nup98 was found also under arsenite stress, whereas Nup153 was not found in SGs (Supplementary Figure S5), suggesting that each stress affects different sets of proteins.

**Figure 8.**
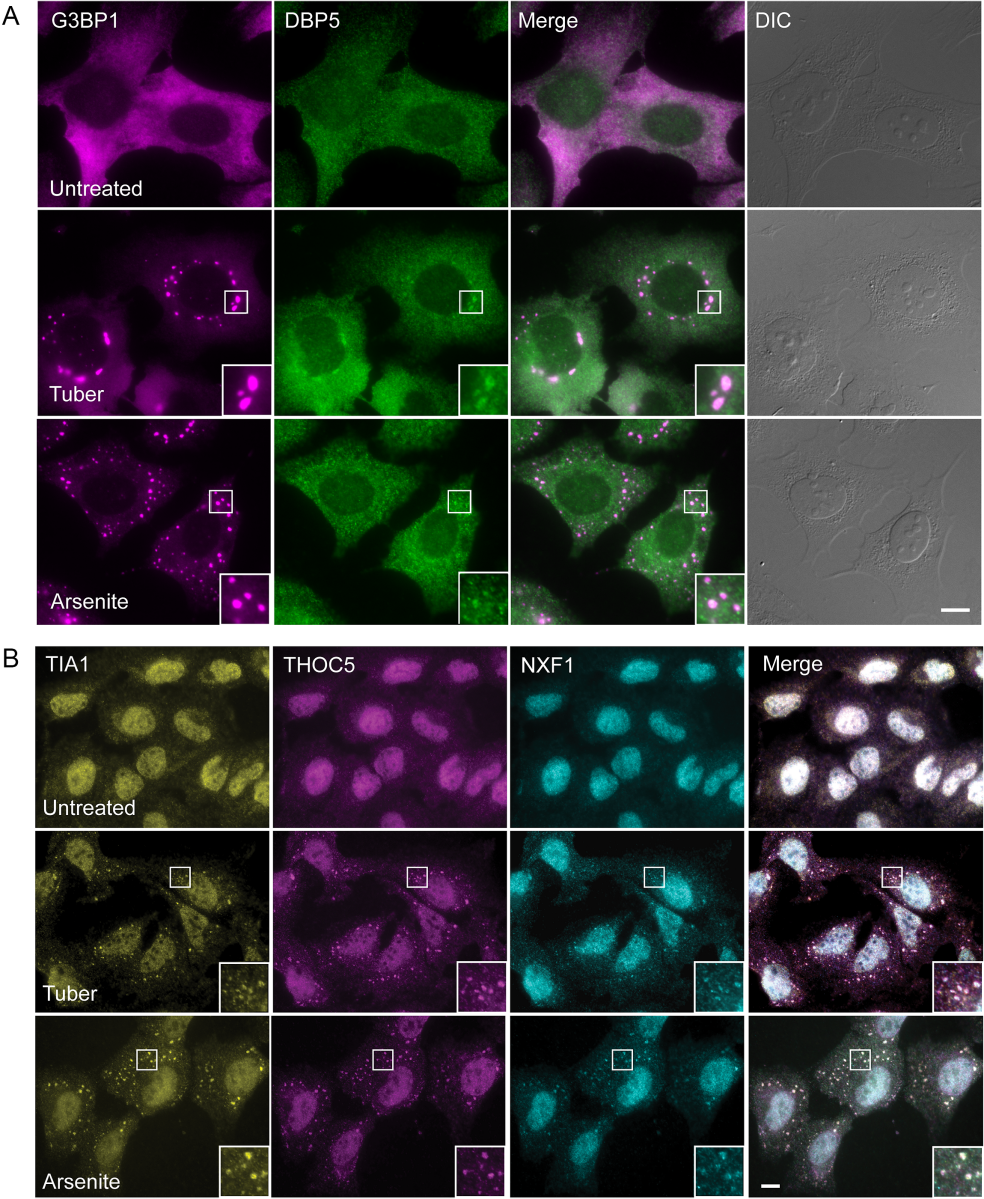
Factors involved in mRNA export accumulate in SGs during tubercidin stress. (**A**) The Dbp5/DDX19 helicase and (**B**) NXF1/TAP and THOC5 are found in SGs during tubercidin stress. This is seen also for NXF1/TAP and THOC5 with arsenite treatment. SGs are labeled with antibodies to G3BP1 and TIA-1. Boxed areas in the images are shown as enlarged boxes. Bar = 10 μm.

Since the Nups on the cytoplasmic side of the NPC that are important for mRNA export did not seem to be mislocalized to SGs, we examined whether the Dbp5/DDX19 helicase, situated on the cytoplasmic side at Nup214, and that acts in mRNA release into the cytoplasm, might be mislocalized. Indeed, Dbp5/DDX19 was found in SGs under tubercidin and arsenite conditions (Figure 8A). Dbp5/DDX19 releases the mRNA export factor NXF1/Tap from the mRNA thus allowing the return of NXF1/Tap into the nucleus (23,43,44). Therefore, we examined if NXF1/Tap and other mRNA export factors might be ending up in SGs. The TREX (TRanscription-EXport) complex (45) recruits the export factor NXF1/Tap to cap-proximal sites and SR proteins recruit NXF1/Tap in downstream regions of the mRNA (4,46,47). NXF1/Tap and the TREX factors THOC5 and THOC6 were found in SGs (Figure 8B and Supplementary Figure S6).

**Figure 9.**
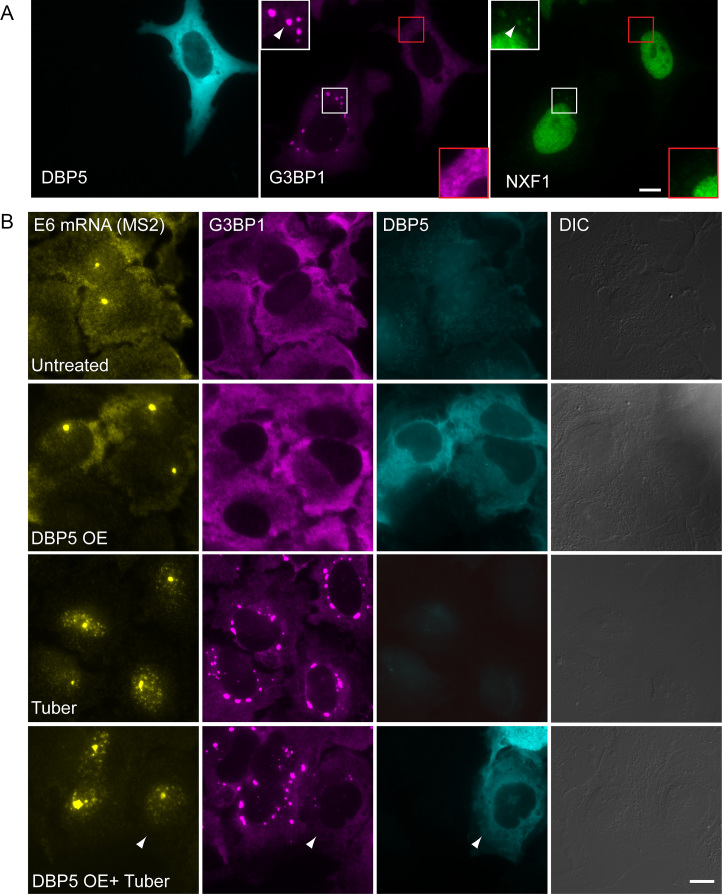
Tubercidin causes an mRNA export impairment even when SGs cannot assemble. (**A**) Overexpression of GFP-Dbp5 (cyan) in cells stops SG formation and NXF1/TAP does not accumulate in the cytoplasm. Boxed areas in the images are shown as enlarged boxes. (**B**) The export of the E6 mRNA, detected by RNA FISH (yellow), is impaired in tubercidin treated cells even when SGs (magenta) do not form due to GFP-Dbp5 overexpression. SGs are labeled with antibodies to G3BP1. Bar = 10 μm.

**Figure 10.**
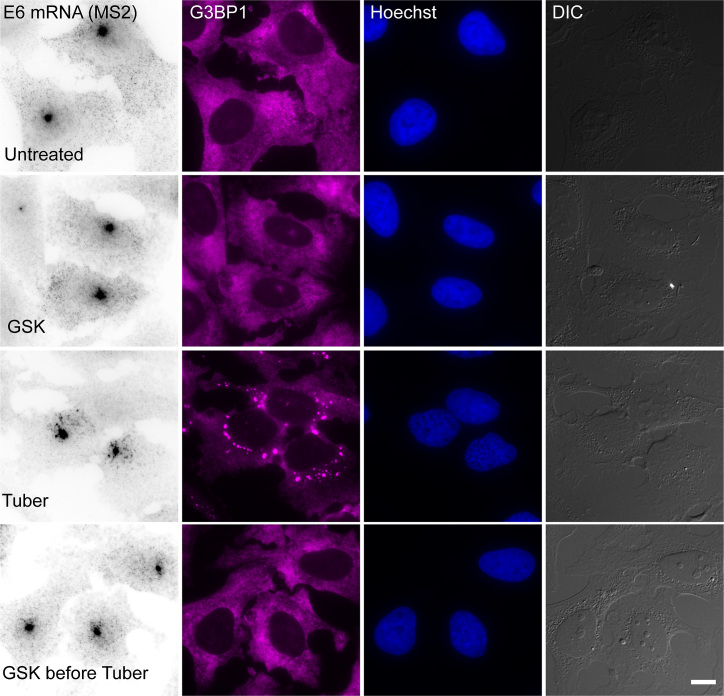
Tubercidin causes an mRNA export impairment even when SGs cannot assemble due to a GSK inhibitor. U2OS cells treated with a GSK inhibitor for 3 h before dox induction for 6 h with or without tubercidin addition. The export of the E6 mRNA, detected by RNA FISH (black), is impaired in tubercidin cells even when SGs (magenta) do not form due to GSK. SGs are labeled with antibodies to G3BP1. Bar = 10 μm.

We next wanted to explore whether the process of SG formation might be the reason for the impairment of mRNA export caused by the stresses. During this study, we found that the overexpression of Dbp5 causes the disassembly of SGs even when the cells were stressed. In this case NXF1 (and THOC6, not shown) were not mislocalized (Figure 9A). However, when we examined the distribution of the E6 mRNA under these conditions, the mRNA was still retained in the nucleus even though SGs had not formed (Figure 9B). Another approach to prevent SGs assembly is inhibition of the kinase PERK that phosphorylates eIF2α using GSK2606414 (48). Cells that were treated with GSK 3 hrs before tubercidin addition together with dox induction for 6 hrs, did not exhibit SG formation and the E6 mRNAs were still stuck in the nucleus (Figure 10). Moreover, proteins involved in nuclear export such as Nup153, NXF1 and THOC5, did not accumulate in the stress granules under those conditions (Supplementary Figure S7). These data demonstrate that the formation of SGs is not the sole reason for the mRNA export impairment, and suggests that stress might cause the relocalization of a variety of factors involved in several mRNA-related processes, such that stress leads to a widespread mislocalization of proteins both in the nucleus and in the cytoplasm, bringing the flow of gene expression to a halt. We therefore examined whether other nuclear processes related to mRNA might be affected.

### Tubericidin interferes with the recruitment of EJC proteins to transcripts and affects nuclear speckle composition

**Figure 11.**
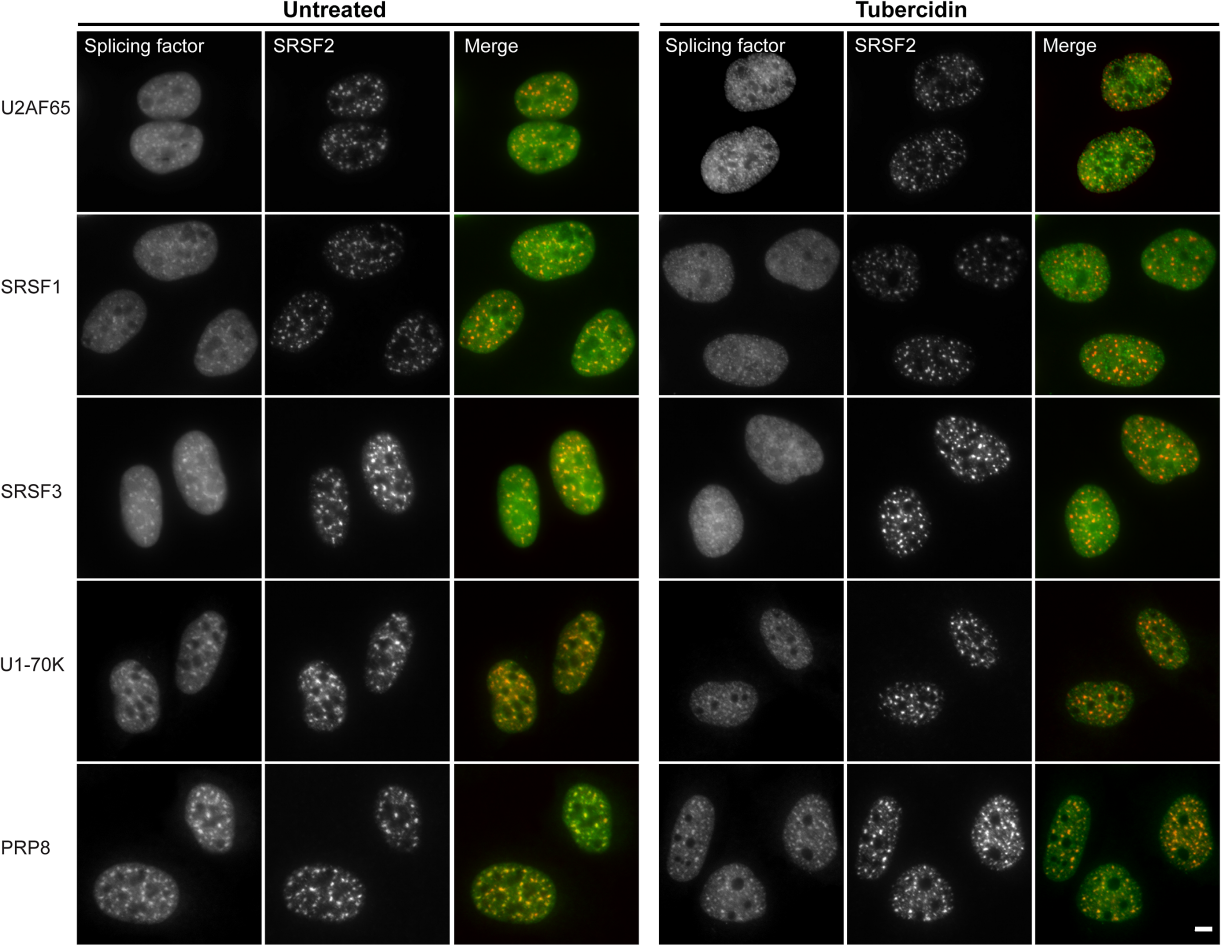
The distribution of splicing factors in nuclear speckles during tubercidin treatment and in untreated cells. The distribution of various GFP-tagged splicing factors stably expressed from BACs under tubercidin treated (6 h) conditions and in untreated cells. Merge: BAC-expressed splicing factors in green and SRSF2 in red. Bar = 5 μm.

As mentioned above, previous work has shown that tubercidin leads to the dispersal of SRSF1 from nuclear speckles while SRSF2 remains in them (14), implying that the each component of nuclear speckles may behave uniquely with respect to nuclear speckle localization. To broadly examine the fate of RBP localization in nuclear speckles under tubercidin conditions, we used GFP-fused splicing factors expressed from stably integrated bacterial artificial chromosomes (BACs) containing the full gene-body of several splicing factors. The genes were expressed at near physiological levels under the control of their endogenous promoters with an in-frame, C-terminal GFP-tag (26,27). In untreated cells, each of these splicing factors accumulated in nuclear speckles with differing efficiencies (Figure 11 and Supplementary Figure S8). After tubercidin treatment, SRSF2, SRSF4, SRSF5, SRSF6, SRSF7 and Prp8 remained in nuclear speckles, whereas SRSF1, SRSF3, U1-70K and U2AF65 were mostly nucleoplasmic (Figure 11 and Supplementary Figure S8). This suggests that the affinity of different splicing factors for nuclear speckle binding sites varies, and that nuclear speckle structure can be maintained even though several protein components are missing.

**Figure 12.**
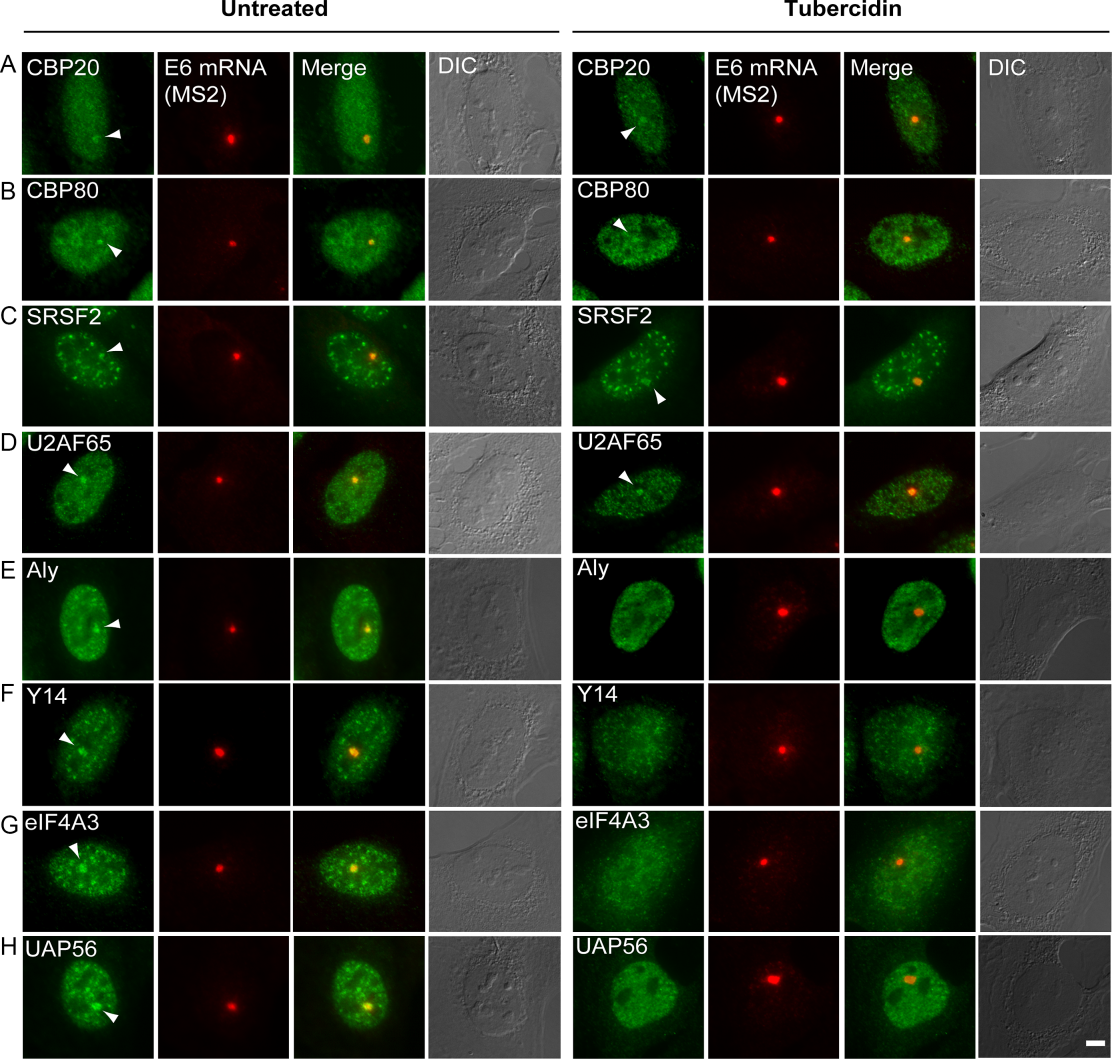
Tubericidin interferes with the recruitment of EJC proteins to transcripts. The recruitment of various RNA processing factors to the transcribing E6 gene was examined by immunofluorescence with antibodies to: CBP20, CBP80, SRSF2, U2AF65, ALYREF, eIF4A3, Y14 and UAP56 (green). The active E6 transcription site was detected by RNA FISH with a Cy5-labeled probe to the MS2 region of the E6 mRNA (red). Arrowheads point to the factors recruited to the actively transcribing genes. Bar = 5 μm.

Next, we reasoned that tubercidin stress might negatively affect the recruitment of other RNA-binding proteins to the mRNA to determine the fate of the mRNAs already at the stage transcription. This was tested using a protein factor recruitment assay performed on the E6 inducible gene (25,49–51). Since the E6 gene was stably integrated as a tandem gene array in U2OS cells, the transcribing array recruits many RNA-binding proteins involved in mRNA processing that can be easily identified over the nucleoplasmic background. Under these conditions, recruitment of the cap binding proteins (CBP20 and CBP80) that are important for mRNA export persisted (Figure 12A, B). Splicing factors were also recruited (Figure 12C, D). Despite the fact that tubercidin is an adenosine analogue, we found that the proteins related to poly(A) processing of mRNA, such as mammalian cleavage factor I subunits CF I_m_68 and CF I_m_25 (22) and the nuclear poly(A) binding protein (PABPN), were recruited to the mRNAs (Supplementary Figure S9).

**Figure 13.**
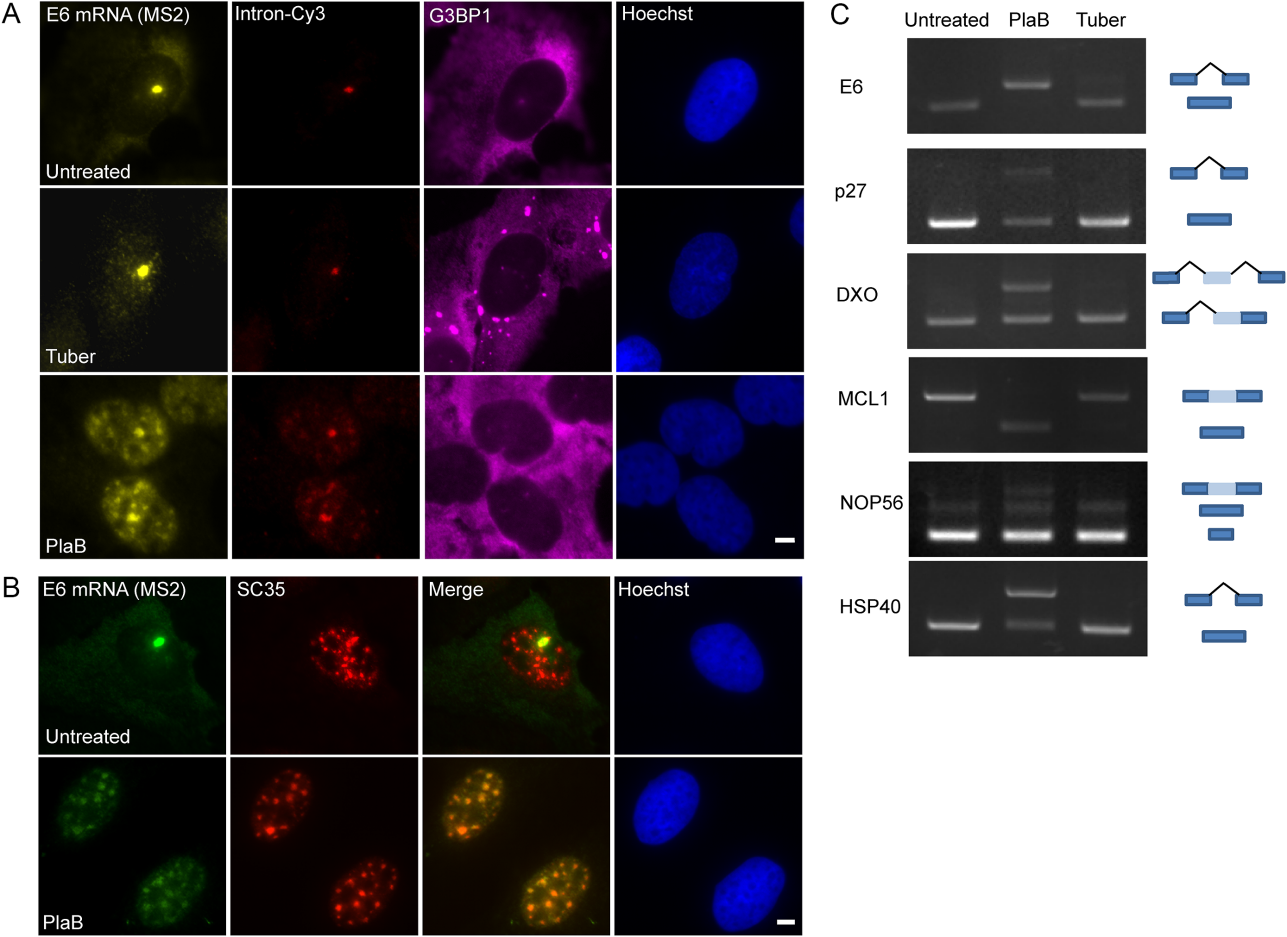
Tubercidin causes an mRNA export defect. (**A**) RNA FISH experiment to detect the distribution of the E6 mRNA in U2OS cells treated with tubercidin and Pladienolide B (PlaB) using a Cy5 labeled probe that detects the MS2 region of the E6 mRNA (yellow), and a Cy3-labeled probe that detects the intron of the E6 mini-gene (red). SGs were detected using an anti-G3BP1 antibody (magenta). Hoechst DNA stain is in blue. (**B**) Splicing inhibition causes RNA retention in nuclear speckles. The localization of E6 transcripts under splicing inhibition by Pladienolide B treatment in U2OS cell was followed by RNA FISH using a Cy5-labeled MS2 probe (green). Nuclear speckles were detected using anti-SRSF2 (red). Hoechst DNA stain is in blue. Bar = 5 μm. (**C**) Semi-quantitative RT-PCR analysis of cells treated with Pladienolide B (10 μM) or tubercidin (10 μM) together with dox induction for 6 h. Cells were examined for intron inclusion of HSP40 and DXO pre-mRNAs and for exon skipping of E6, MCL1, NOP56 and p27 pre-mRNAs. The positions of different cDNA products are noted on the right of the gel images.

In light of the changes in the splicing factors nuclear distribution under tubercidin treatment (Figure 11), we next examined if RNA splicing patterns might be changing upon tubercidin stress. When RNA FISH for the E6 mRNA was performed using the MS2 probe and compared to a probe that detects the E6 intron, the intronic signal was found only at the transcription site (Figure 13A), suggesting that splicing is not inhibited by tubercidin. This was in contrast to treatment with Pladienolide B, a splicing inhibitor (52), which caused unspliced E6 mRNAs (detected with the intron probe) to accumulate in nuclear speckles during the export impairment (Figure 13B). It is known that non-spliced mRNAs accumulate in the nuclear speckles when pre-mRNA splicing is inhibited (25,53–56). Interestingly, Pladienolide B did not induce SG formation, demonstrating that the two drugs act on different pathways. Moreover, examining the patterns of alternative splicing of several genes, as performed in (57), showed that tubercidin did not change splicing patterns whereas the Pladienolide B splicing inhibitor did (Figure 13C).

In contrast to the above RBPs that were co-transcriptionally recruited to the active transcription sites under tubercidin stress (Figure 12A-D), accumulation of the export factor ALYREF at the E6 transcription site was significantly reduced (Figure 12E). ALYREF is also part of the TREX complex but this factor did not accumulate in SGs upon stress. Additionally, there was reduced recruitment of the exon junction complex (EJC) proteins eIF4A3, Y14 and UAP65 to the active genes (Figure 12F–H). These factors function in nuclear mRNP metabolism with connections to splicing, mRNP packaging and perhaps to export of some mRNA species (58). We noticed that the EJC proteins that were not recruited to the mRNAs transcribed on the E6 transcription sites were nucleoplasmically dispersed rather than being present in nuclear speckles, in contrast to some of the splicing factors that remained in the speckles under tubercidin treatment (Figure 12), and in contrast to the E6 mRNAs that significantly accumulated in the nuclear speckles (Supplementary Figure S3). This meant that a) the E6 transcripts are not coated by these EJC proteins, and b) nuclear speckles still formed even though some components such as EJC factors were lacking within them. This complements the information obtained above on differential splicing factor interactions with nuclear speckles (Figure 11), and the nucleoplasmic dispersal of MALAT1 lncRNA (Figure 3). Altogether, we find that stress leads to the severing of the nucleo-cytoplasmic distribution of RBPs such that key factors are mislocalized, thereby ending up in an obstruction to mRNA export.

## DISCUSSION

The stress pathway induced by tubercidin uncouples nuclear RNA metabolism from cytoplasmic RNA metabolism, thereby separating the compartments. The normal flow of the gene expression pathway is blocked during this stress response even though transcription persists, leading to the mislocalization of RNAs. Hence, tubercidin is toxic to all cell types. It failed as a chemotherapeutic agent even though it is one of several adenosine analogues, many of which are used to target cancer cells (59,60). A phase I trial in which tubercidin was administered to cancer patients showed that it caused severe effects in some human tissues, as well as venous thrombosis and necrosis of the tissue at the site of injection. Taking these effects into consideration, another phase I trial was conducted but concluded that nephrotoxicity and local irritation of the veins were too severe, and the overall effect on tumor progression was limited (19,20). Therefore, tubercidin is not administered to patients for the treatment of cancer. Our study shows why tubercidin could not be used systemically on humans. Tubercidin does not preferentially target cancer cells as anticipated, rather, it negatively affects RNA metabolism processes in both normal and cancer cells, and its effects are irreversible (not shown). Global changes to the cells are not observed after a few hours of treatment as seen in the DIC images. In the nucleus, even though transcription persists, tubercidin hampers the co-transcriptional recruitment of TREX and EJC proteins to spliced mRNAs, disrupts nuclear speckle structure and the balance of protein distribution in the nucleoplasm, mislocalizes the lncRNA from speckles, and impairs nucleo-cytoplasmic mRNA export. Future studies will be required to examine if the export of other types of RNAs such as rRNAs in pre-ribosomal subunits are also affected. In the cytoplasm, tubercidin induces a protein translation stress pathway that induces stress granule formation. These granules accumulate several proteins that are crucial in the mRNA export pathway – nucleoporins and mRNA export adaptors – such that altogether the balance between the compartments is toppled.

We found a redistribution of RNAs with respect to nuclear speckles, namely, the lncRNA MALAT1 usually found in nuclear speckles was dispersed in the nucleoplasm whereas spliced mRNAs accumulated in nuclear speckles. This exemplifies the incorrect balance within the nuclear compartment. Possibly, the entry of these mRNAs into nuclear speckles causes the displacement of MALAT1. It is known that mRNAs can pass through nuclear speckles at diffusion rates that are similar to the nucleoplasm but normally mRNAs do not amass within nuclear speckles (61–64). The accumulation of export-deficient mRNAs in nuclear speckles has been demonstrated in several studies (25,54,55,65–67), but notably, this was observed for unspliced intron-containing pre-mRNAs. Also, it was shown that EJC proteins are not recruited to transcription sites of splicing defective mRNAs (53). Here we show that spliced RNAs that do not contain EJC proteins can also accumulate in nuclear speckles, and that EJC proteins that usually reside within nuclear speckles (68), are dispersed in the nucleoplasm. The observations that export defective mRNAs, either intron-containing mRNAs or spliced mRNA without EJC, accumulate in nuclear speckles, strengthen the suggestion that mRNAs can pass through the nuclear speckles as they travel in the nucleoplasm and prior to mRNA export (55,69), and their interactions with nuclear speckles might be enhanced under defective conditions. Hence, one speculation could be that nuclear speckles can serve as a quality check station for mRNAs before export.

The outcome of tubercidin stress allows us to come to several conclusions regarding the composition and fidelity of nuclear speckles. First, nuclear speckle structures can persist even when the following nuclear speckle factors became mostly nucleoplasmic: splicing factors—SRSF1, SRSF3, U1–70K and U2AF65; the ALYREF TREX factor; EJC factors—eIFA3, Y14 and UAP65; and 3′-end factors CF I_m_68, CF I_m_25 and PABPN. The splicing factors that remained in nuclear speckles and therefore might be considered ‘core’ nuclear speckle factors were SRSF2, SRSF4, SRSF5, SRSF6, SRSF7 and Prp8. Indeed, it was recently shown that different proteins occupy different sub-regions within nuclear speckles (70). Second, the MALAT1 and NEAT1 lncRNAs were not required for nuclear speckle maintenance. A previous study has demonstrated that SRSF1 and MALAT1 are important for nuclear speckle assembly, as seen by depleting these components (71). Altogether, these studies suggest that nuclear speckle assembly and nuclear speckle maintenance utilize different core factors. The uniqueness of tubercidin treatment compared to studies where one or two factors were depleted, is that tubercidin treatment shows that the nuclear speckle structure can persist even though 13 protein and RNA components that we tested were simultaneously lacking or dramatically reduced in nuclear speckles.

The results also suggest that tubercidin as an adenosine analogue does not stop transcription. It probably does not obstruct the capping of the mRNA as seen by the recruitment of capping protein, possibly because guanosine is the capping nucleotide. The splicing reaction also continued as observed in an RNA FISH experiment with a probe to an intron showing that the nucleoplasmic mRNA did not contain the intron, by RT-PCR and by the continued recruitment of splicing factors to the induced gene. It seems therefore that this stress pathway which does not drastically disrupt transcription and splicing, is targeted by the cell at the mRNA export level and also involves the redistribution of nuclear proteins into cytoplasmic stress granules.

Stress granules form in the cytoplasm of eukaryotic cells upon a variety of stresses including include oxidative stress, heat-shock, viral infection, and proteosome inhibition. SGs contain untranslating mRNPs stalled at the stage of translation initiation (31,72), and many proteins, in particular RNA-binding proteins (73). SGs can form through a pathway that requires the phosphorylation of the α-subunit of the translation initiation factor eIF2 (eIF2α) at residue Ser51, which reduces the levels of the eIF2.GTP.Met-tRNAi^Met^ ternary complex that is required for translation initiation. We found that tubercidin leads to the phosphorylation of eIF2α, but with slower kinetics in comparison to arsenite that is commonly used to induce SG formation. While the presence of RBPs in SGs is integral to SG formation, the localization of NPC subunits and mRNA export factors within SGs was unexpected. Recently, two studies have found that nucleoporins and transport factors are targeted to SGs during arsenite- or sorbitol-mediated stress (74,75). One study showed that arsenite and sorbitol disrupt protein import and export by causing the accumulation of Ran, karyopherins, and Nups in SGs. We however, found that tubercidin stress affects mRNA export (75). Yet, the accumulation of export factors in SGs is not necessarily the direct reason for the mRNA export impairment, since inhibiting the formation of SGs did not release the obstruction. Also, the NPC structures still existed even though some fraction of Nups accumulated in SGs under tubercidin treatment. The Dbp5/DDX19 helicase that assists in releasing mRNA from the NPC is also in SGs, as are the major mRNA export factor NXF1/Tap and some TREX components. It therefore seems reasonable that the many changes occurring in the nucleus in RNP formation are together rendering the mRNA export-incompetent. Taken together, this study shows that when imposed with stress that does not stop mRNA transcription, the cell ensures by a variety of means that the mRNA does not reach the translation machinery.

## Supplementary Material

Supplementary Data
