# Supplementary material for: Uncoupling of nucleo-cytoplasmic RNA export and localization during stress
Source: Nucleic Acids Res. 2019 Mar 13;47(9):4778–97. doi: 10.1093/nar/gkz168 (PMC6511838; doi:10.1093/nar/gkz168)
Supplement: Supplementary Data [file gkz168_supplemental_files.zip › Supp Hochberg et al revised final.pdf]

Supplemental Material

Uncoupling of nucleo-cytoplasmic RNA export and localization during stress

Hodaya Hochberg-Laufer[1], Avital Schwed-Gross[1], Karla M. Neugebauer[2]

and Yaron Shav-Tal[1*]

Supplemental figures

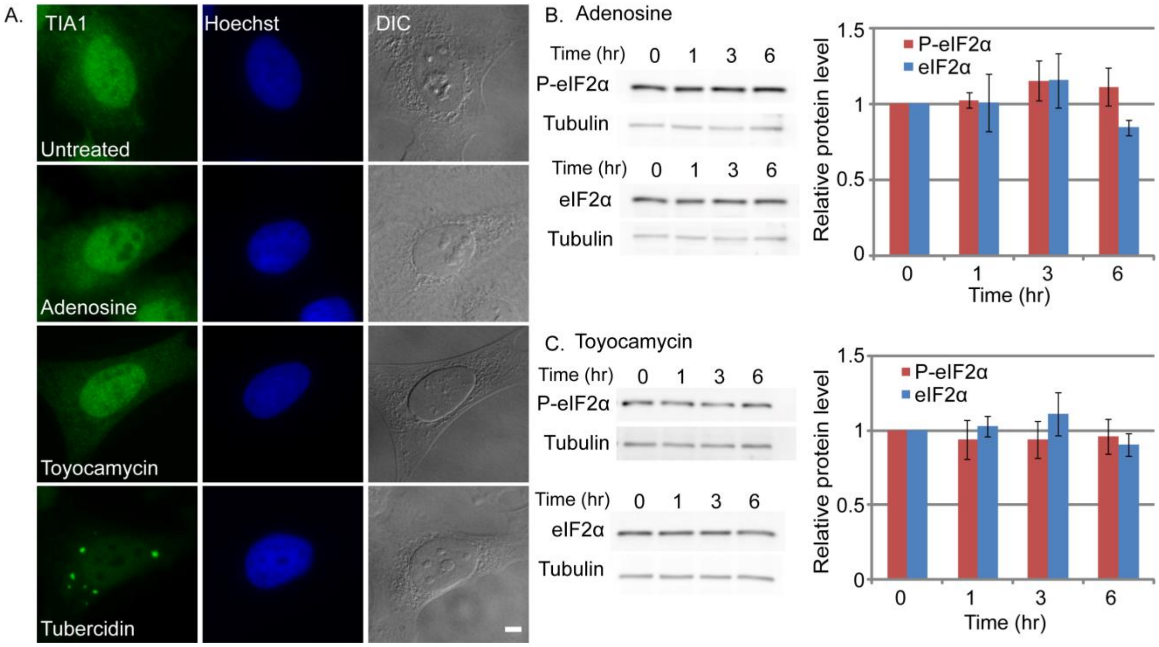

Figure S1. Toyocamycin and adenosine do lead to stress granules formation and have no effect on the phosphorylation levels of eIF2α. (A) U2OS cells treated with 10 μM of adenosine, toyocamycin or tubercidin for 6 hrs shows stress granule formation (green) only under tubercidin treatment. SGs were identified with an antibody to TIA-1. Hoechst DNA stain is in blue. DIC in grey. Bar = 5 μm. (B) Western blot analysis of eIF2α and phosphorylated eIF2α protein levels in U2OS cells after adenosine or (C) toyocamycin treatments for different times. Tubulin used as a loading control. Blots are representative of 3 independent experiments. The average quantification of 3 repeated experiments is presented in the plots (mean ± SEM). There were no significant differences in the relative levels of phosphorylated eIF2α between the time point 0 hrs and the treatments in cells treated with adenosine (one sample t-test, 1hr p=0.7018, 3 hr p=0.3862 and 6 hr p=0.4695) or in cells treated with toyocamycin (one sample t-test,

1 hr p=0.5836, 3 hr p=0.562 and 6 hr p=0.6314). Similarly, there were no significant differences in the relative levels of eIF2α between the time point 0 hrs and the treatments in cells treated with adenosine (one sample t-test, 1 hr p=0.9118, 3 hr p=0.4958 and 6 hr p=0.09972) or in cells treated with toyocamycin (one sample t-test, 1 hr p=0.7547, 3 hr p=0.575 and 6 hr p=0.2768).

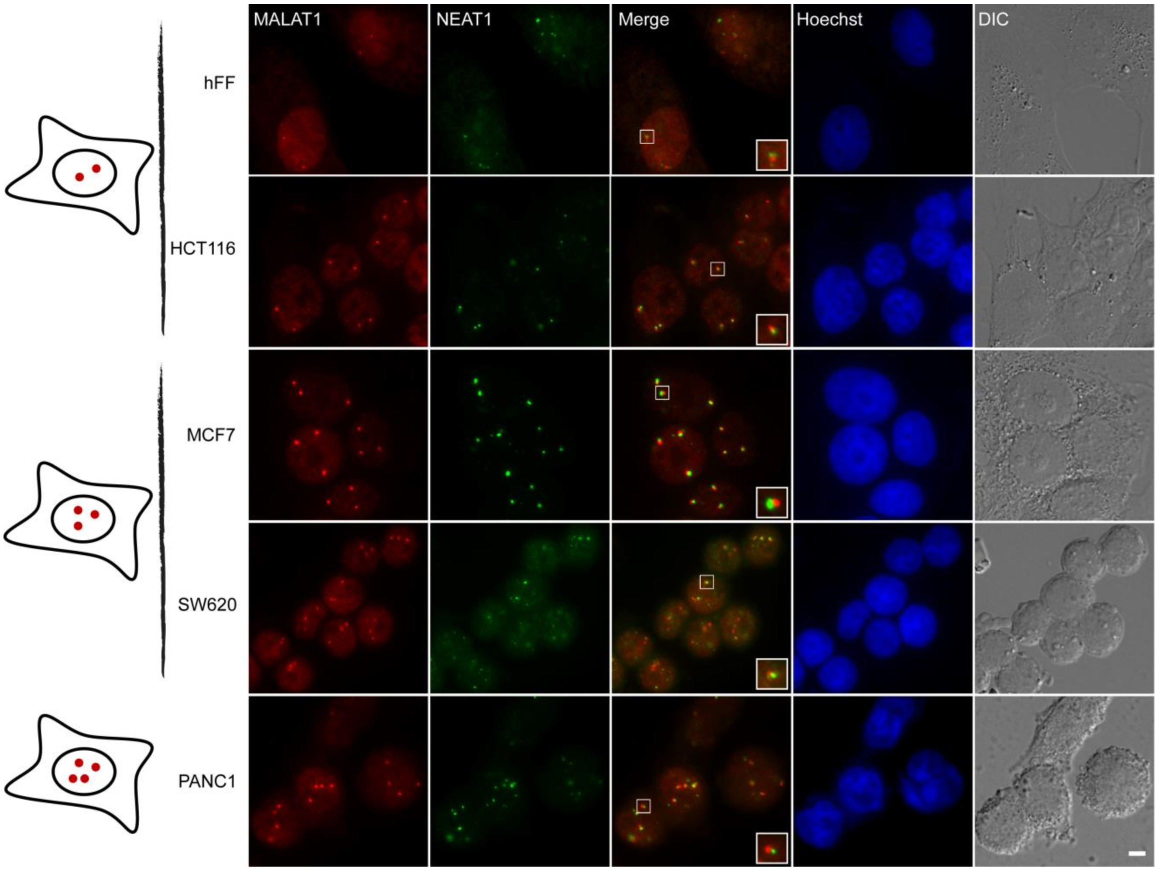

Figure S2. Tubericidin does not interfere with MALAT1 and NEAT1 transcription in different cell types. RNA FISH with probes to MALAT1 (red) and NEAT1 (green) show the distribution of these lncRNAs in hFF, HCT116, MCF7, SW620 and PANC1 cell lines. Hoechst DNA stain is in blue. Boxed regions are shown as enlarged boxes. DIC in grey. Bar = 5 µm.

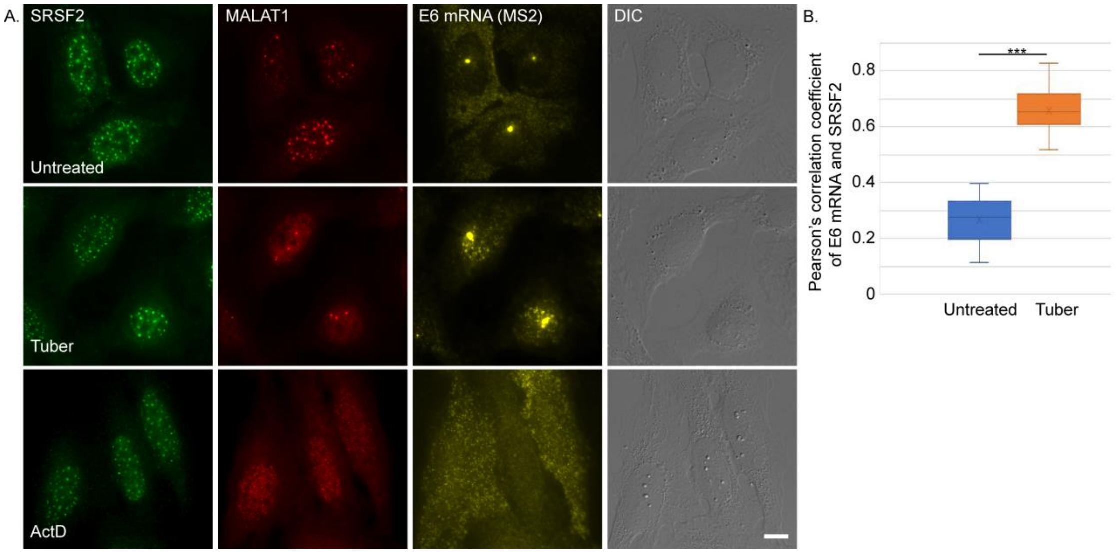

Figure S3. (A) MALAT1 foci detected after tubercidin treatment represent transcription sites. The transcription sites of E6 and MALAT1 detected by RNA FISH with a Cy5-labeled MS2 probe (for E6, yellow) and a MALAT1 (Cy3, red) in U2OS cells under treatment with tubercidin (6 hrs) and actinomycin D (2 hrs, 5 µg/ml). Nuclear speckles were detected using an SRSF2 antibody (green). Bar = 10 µm. (B) Pearson's correlation coefficient measuring the colocalization of the E6 mRNA and SRSF2 in speckles was significantly higher (one sample t-test, ****P* < 0.001) in cells treated with tubercidin (Rr=0.66) than in untreated cells (Rr=0.27). The box plot represents data from 50 different cells.

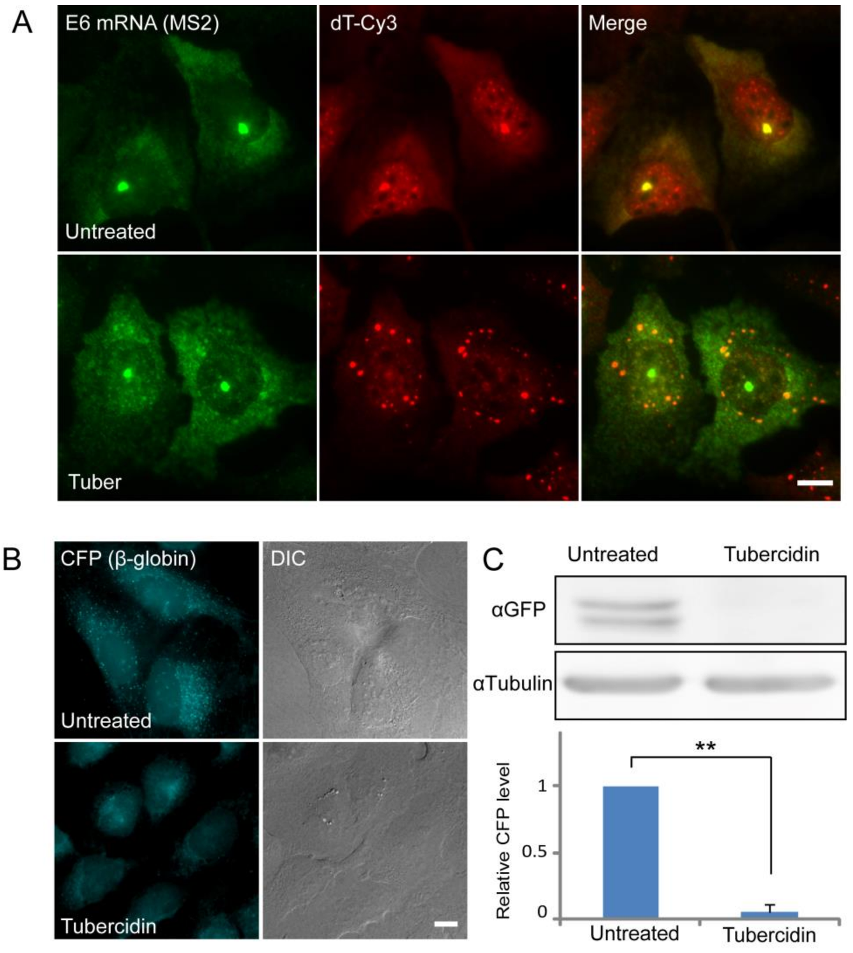

Figure S4. E6 transcripts accumulate in SGs under tubercidin conditions. (A) The E6 mRNA (RNA FISH with an MS2 probe, green) was detected in SGs (RNA FISH with an oligo-dT probe, red) under tubercidin treatment conditions (6 hrs). Bar = 10 µm. (B) Tubercidin inhibits the translation of the CFP protein expressed from the E6 gene. The CFP-SKL protein encoded by the E6 gene is targeted to cytoplasmic peroxisomes in dox-induced cells (top, cyan dots), whereas tubercidin treatment reduces the CFP fluorescence in the cells (bottom). Bar = 10 µm. (C) Western blot analysis with an anti-

GFP antibody that detects CFP-SKL protein levels in E6 cells in untreated cells and after tubercidin treatment (6 hrs). Tubulin used as loading control. The average quantification was based on 3 independents experiments (mean ± sd). A two-tailed *t* test was performed. ** $P < 0.01$.

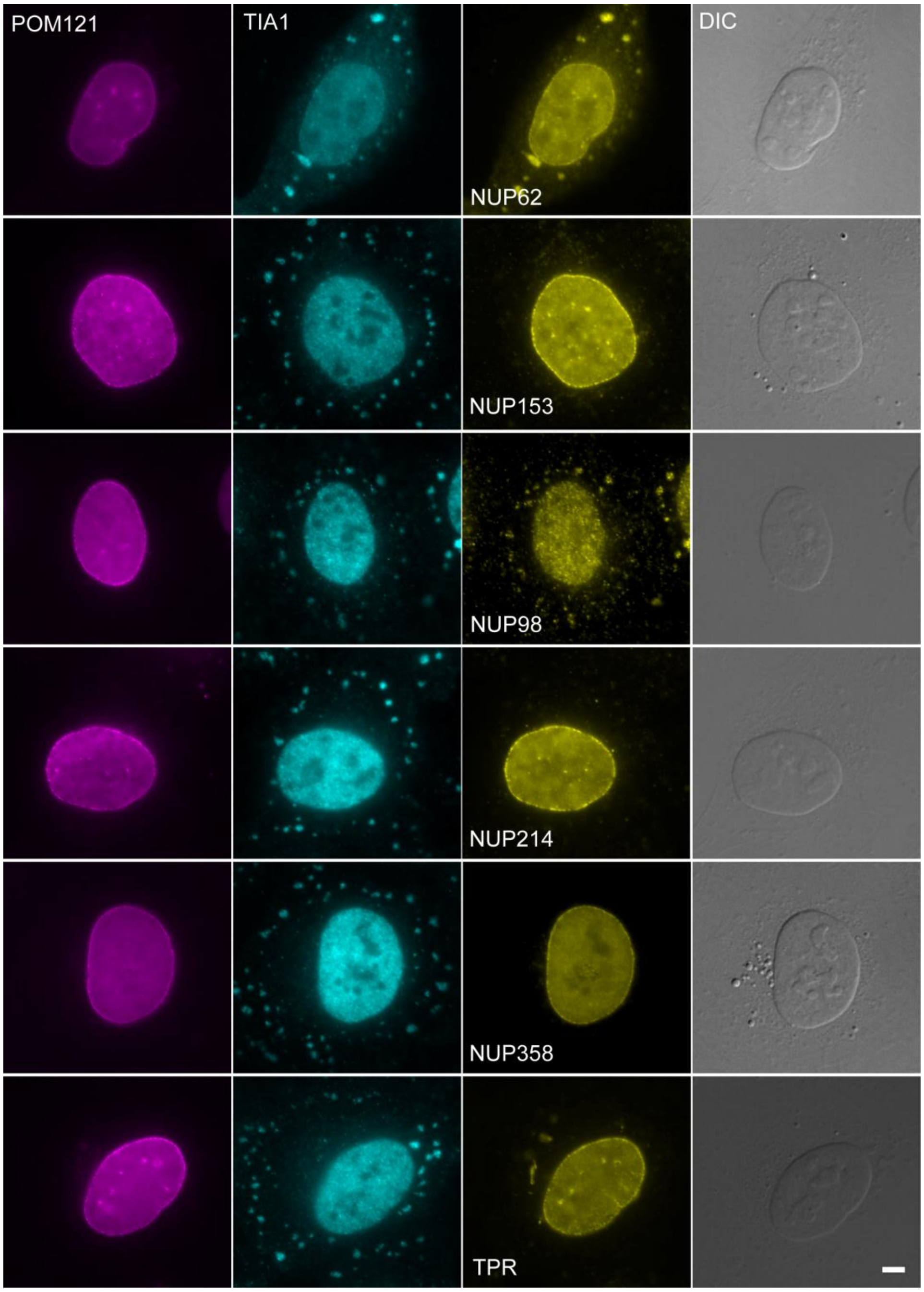

Figure S5. Nucleoporins accumulate in SGs during arsenite stress. Arsenite stress (45 min) causes the accumulation of some nucleoporins in SGs. POM121 nucleoporin that does not change during stress was used as a control to demarcate the NPCs. SGs were identified with an antibody to TIA-1. Bar = 5 µm.

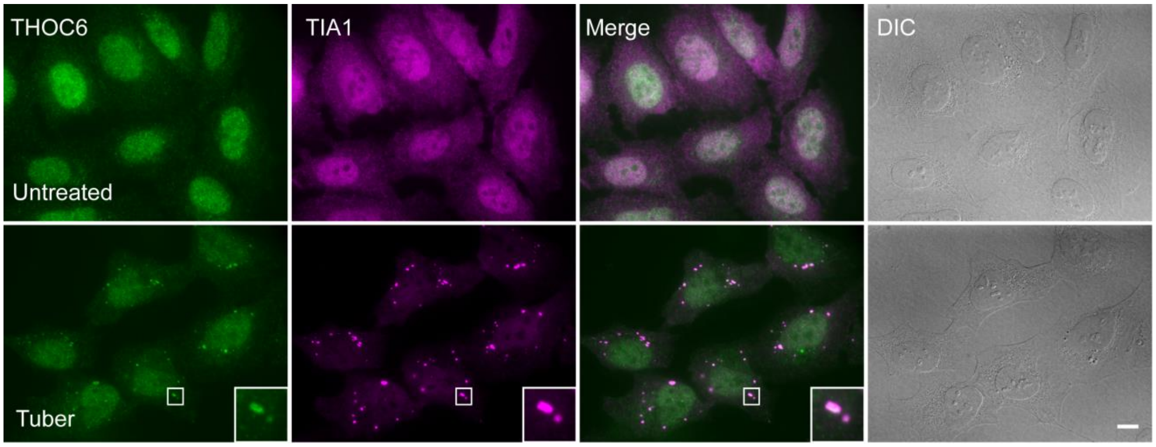

Figure S6. Factors involved in mRNA export accumulate in SGs during tubercidin stress. THOC6 (green) is found in SGs (magenta) during tubercidin stress. SGs are labeled with antibodies to TIA-1. Bar = 10 µm.

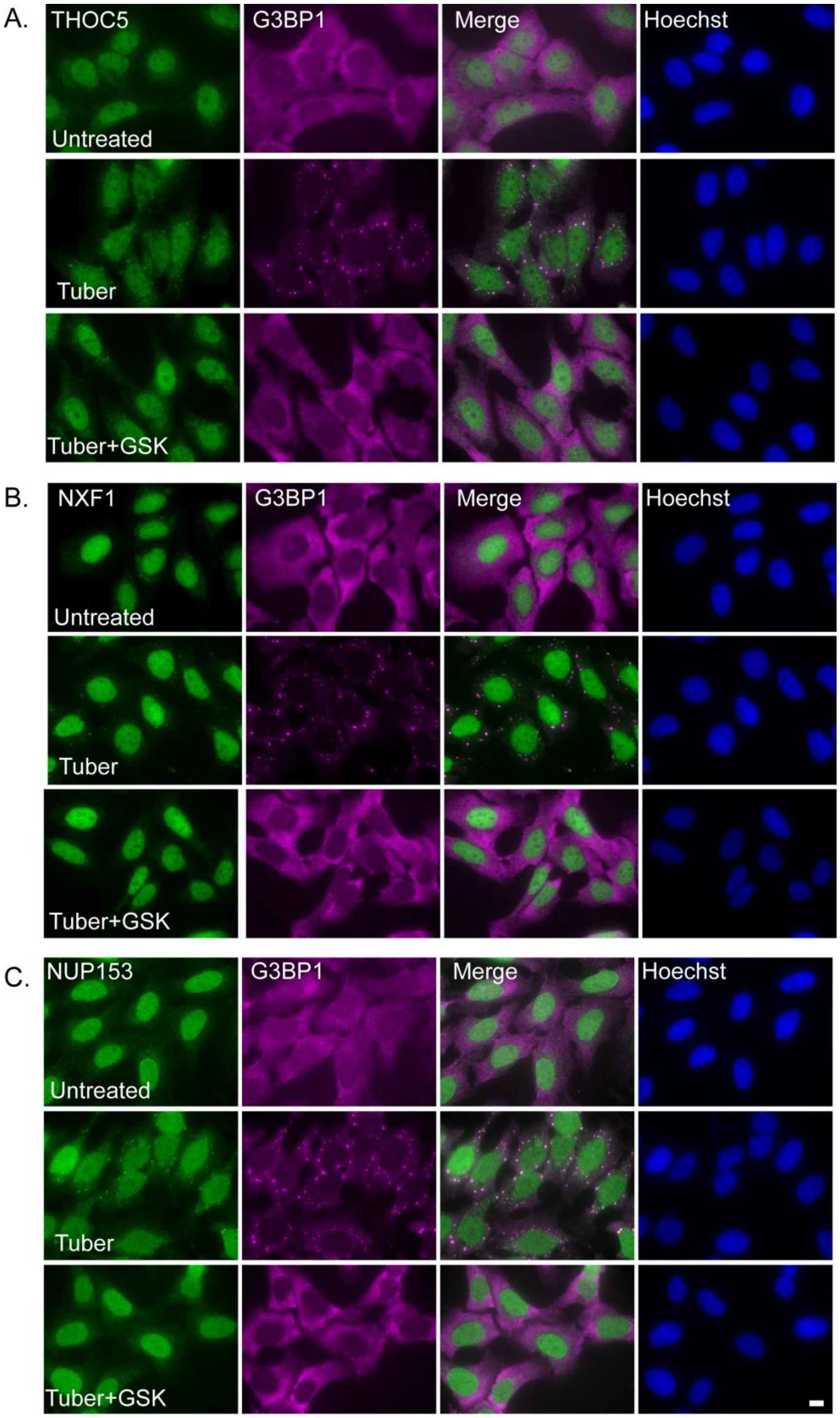

Figure S7. Factors involved in nuclear export do not accumulate in the stress granules under tubercidin treatment together with GSK. The accumulation of (A) THOC5, (B) NXF1 and (C) Nup153 (green) in stress granules (magenta) was examined in U2OS cells treated with tubercidin only or together with GSK**.** Hoechst DNA stain is in blue. Bar = 10 µm.

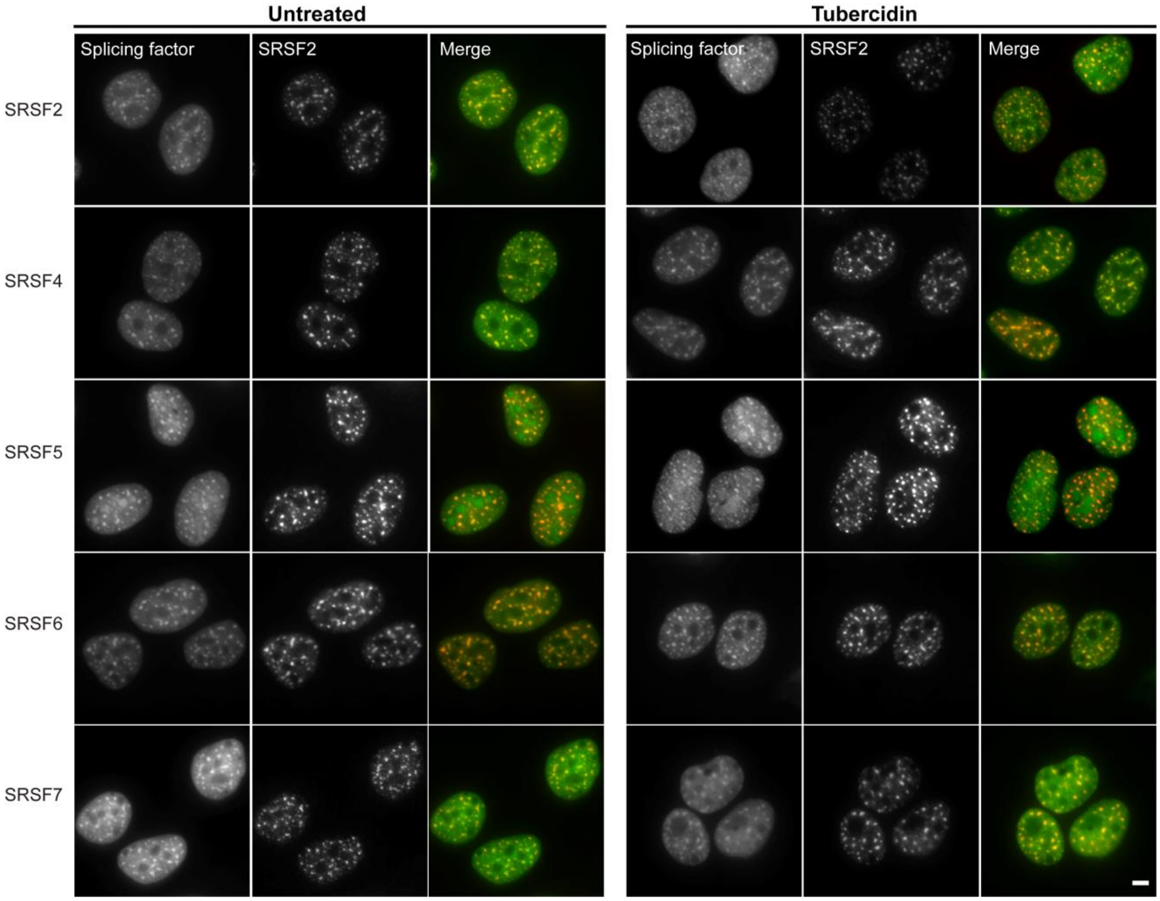

Figure S8. The distribution of splicing factors in nuclear speckles during tubercidin treatment and in untreated cells. The distribution of various GFP-tagged splicing factors stably expressed from BACs under tubercidin treated (6 hr) condition and in untreated cells. Merge: BAC-expressed splicing factors in green and SRSF2 in red. Bar = 5 µm.

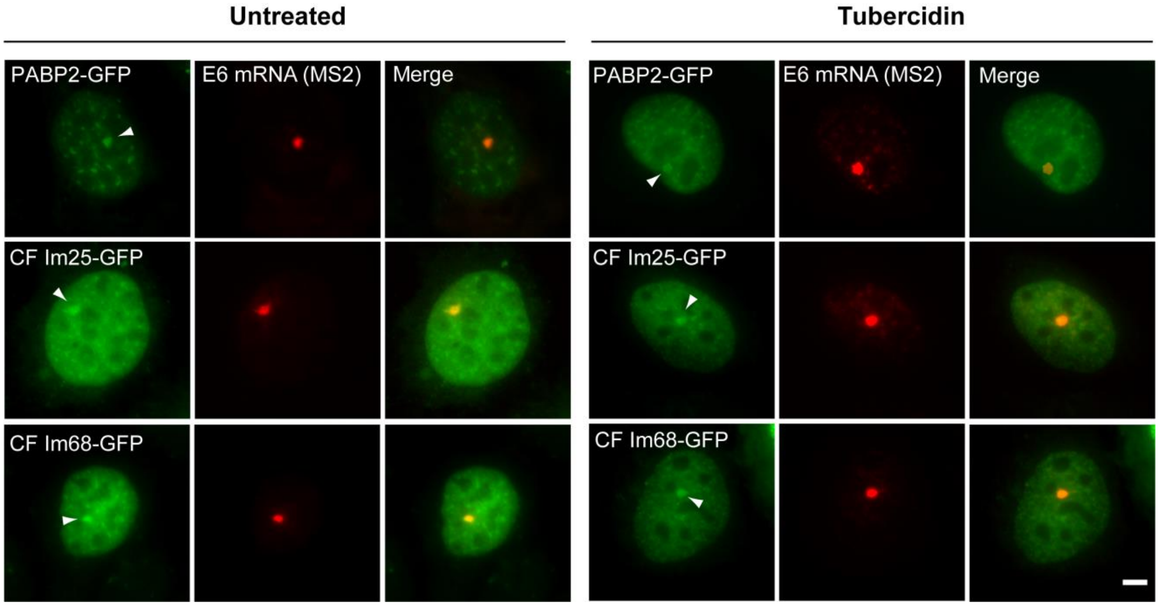

Figure S9. Tubercidin does not affect the recruitment of poly(A) processing factors to the E6 transcription sites. The poly(A) processing factors PABP2-GFP, CF I_m68-GFP and CF I_m25-GFP (green) were transiently transfected in U2OS cells to examine the recruitment of those factors to the E6 transcription sites detected by RNA FISH (MS2 probe, red) under untreated and tubercidin treated (6 hrs) conditions. Arrowheads point to the factors recruited to the actively transcribing genes. Bar = 5 µm.

Supplemental movie legends

Movie S1. Time-lapse imaging showing SG formation in U2OS cells stably expressing GFP-IGF2BP3 under treatment with arsenite. Images were acquired every 15 minutes for 75 min.

Movie S2. Time-lapse imaging showing SG formation in U2OS cells stably expressing GFP-IGF2BP3 under treatment with tubercidin. Images were acquired every 15 minutes for 210 min.
